# Mitochondria as the Hub of Apoptosis: A Comprehensive Insight From Mitochondria to Interactions With Other Organelles

**DOI:** 10.1002/mco2.70911

**Published:** 2026-08-26

**Authors:** Rubin Tan, Jinming Zhao, Rong Wang, Yue Shi, Zaozao Chen, Yixuan Ma, Na Li, Wenqing Duan, Duo Lin, Chunxiang Zhang

**Affiliations:** ^1^ Department of Physiology School of Basic Medical Sciences Southwest Medical University Luzhou China; ^2^ Department of Cardiology Institute of Cardiovascular Research the Affiliated Hospital Southwest Medical University Luzhou China; ^3^ The Key Laboratory of Medical Electrophysiology of the Ministry of Education Institute of Cardiovascular Research Southwest Medical University Luzhou China; ^4^ Department of Pathology College of Basic Medical Sciences China Medical University Shenyang China; ^5^ Department of Pathology The First Hospital of China Medical University Shenyang China; ^6^ Department of Cardiology, West China Hospital Sichuan University Chengdu China; ^7^ Department of Physiology School of Basic Medical Sciences Xuzhou Medical University Xuzhou China; ^8^ College of Life Sciences Xuzhou Medical University Xuzhou China; ^9^ First Clinical Medical School Xuzhou Medical University Xuzhou China

**Keywords:** apoptosis, MAM, mitochondria, mitochondrial autophagy, mitochondrial transplantation, MOMP, mtDNA

## Abstract

Apoptosis is a core program regulating organismal homeostasis and plays a pivotal role in the onset and progression of most diseases. Increasing evidence in recent years indicates that mitochondria are not only central to cellular metabolism but also play a pivotal role in regulating apoptosis. However, no systematic review elucidating how mitochondria finely regulate apoptotic processes through multidimensional mechanisms, including apoptosis‐resistant diseases such as cancer. This paper systematically summarizes the molecular mechanisms by which mitochondria mediate apoptosis. We focus on the regulation of cytochrome *c* (Cyt c) release by the Bcl‐2 protein family and the activation of downstream caspase cascades. Furthermore, we provide an in‐depth analysis of intrinsic factors, including mitochondrial structural remodeling (membrane rupture, cristae remodeling, and membrane lipid redistribution), dynamics imbalance (fusion, fission, and mitophagy), and mitochondrial DNA abnormalities, as well as extrinsic factors involving interorganelle interactions with the endoplasmic reticulum, lysosomes, and other organelles. Additionally, we review clinical and preclinical advances in drugs targeting these pathways. This review aims to provide a comprehensive perspective on the complex network of mitochondrial regulation of apoptosis and offer valuable insights for developing novel clinical therapeutic strategies for cancer and other diseases.

## Graphical abstract

Mitochondria is the hub of apoptosis in various diseases. The disruption of mitochondrial structure (including membrane rupture, cristae remodeling, and mitochondrial membrane lipid redistribution), the imbalance of mitochondrial dynamics (including fusion and fission, autophagy), the release, disruption, and mutation of mitochondria DNA, as well as interactions with other organelles such as the endoplasmic reticulum, nucleus, lysosomes, and lipid droplets, all induce and promote apoptosis.

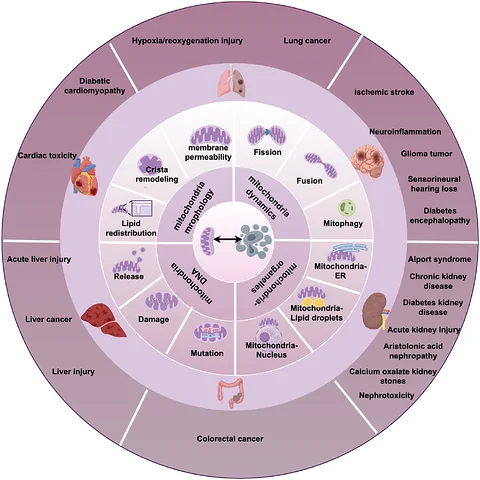

## 1 Introduction

Apoptosis is a programmed cell death with conservation, and its activation can be achieved through exogenous pathways mediated by death receptors and endogenous pathways regulated by mitochondrial pathways and Bcl‐2 [1]. The regulatory mechanism of cell apoptosis is complex, involving multiple organelles and molecular pathways [2]. The endogenous apoptotic pathway is mainly triggered by mitochondrial outer membrane permeabilization (MOMP), leading to the release of cytochrome c (Cyt *c*) and activation of downstream caspase cascade reactions, while the release process of Cyt *c* is strictly regulated by the Bcl‐2 protein family [2]. The exogenous pathway is initiated by the cell membrane protein of the death receptor, which binds to the corresponding ligand and recruits adaptor proteins to assemble the death‐inducing signaling complex that activates Caspase‐8/10 and the downstream cell apoptosis cascade [2]. Apoptosis, as an important biological process for maintaining homeostasis in the body, is widely involved in physiological activities such as embryonic development, aging regulation, immune response, and clearance of damaged cells [3]. Especially in the process of tumor development, abnormal activation of apoptosis signaling pathways can produce antitumor effects.

As the energy metabolism center (“power workshop”) of cells, mitochondria play a pivotal role in apoptosis regulation. They mediate programmed cell death through intrinsic apoptotic pathways, and the regulatory mechanisms of this process involve multiple levels, such as morphological remodeling, mitochondrial dynamics, DNA damage response, and organelle interactions [4, 5]. In recent years, multiple studies have found that changes in mitochondrial morphology are key to maintaining mitochondrial function and are involved in the release of Cyt *c* [5]. The balanced mitochondrial dynamics, including fusion, fission, and mitochondrial autophagy, ensure mitochondrial function and enable adaptation to metabolic changes or cellular stress. In addition, during the process of cell apoptosis, mitochondrial DNA (mtDNA) can be released into the cytoplasm through the Bax/Bak (also known as Bak1) macropores on the outer membrane of mitochondria, participating in inflammatory reactions [6, 7]. At the same time, damage to mtDNA also participates in promoting cell apoptosis [8, 9]. It is worth noting that significant progress has been made in the study of organelle interactions in recent years, among which the interaction between endoplasmic reticulum (ER) and mitochondria can regulate the transmission of apoptotic signals, providing new insights for the study of molecular mechanisms [10]. Hence, there are complex synergistic effects among multiple regulatory mechanisms, and their spatiotemporal dynamic characteristics and pathophysiological correlations urgently need to be further analyzed.

Although existing research has preliminarily revealed multiple mechanisms by which mitochondria regulate apoptosis, there is still a lack of systematic understanding of how these mechanisms form a dynamic regulatory network through synergistic effects. Therefore, we searched the PubMed database for relevant literature published between 2000 and 2025, using the keywords of “mitochondria” and “apoptosis”. The results indicate that mitochondria mediate apoptosis mainly through MOMP, lipid distribution, cristae remodeling, mtDNA, mitochondrial autophagy, mitochondrial fission, mitochondrial fusion, and crosstalk with other organelles. Based on this, this study aims to systematically elucidate these mechanisms and provide a theoretical basis and novel insights for the development of targeted drugs and precision therapeutic strategies based on mitochondrial homeostasis regulation.

## 2 Mitochondrial Morphology and Structure Contribute to Apoptosis

Mitochondria are not only the “energy factory” of eukaryotic cells, but also the key hub for regulating the fate of cells. For a long time, the core role of mitochondria in cell apoptosis has been widely recognized, and the dynamic changes of its morphology and structure are the important material basis for regulating the signal transmission and execution of apoptosis. In the process of apoptosis, mitochondria not only participate in the reaction as a passive organelle, but also promote the programmed cell death through a series of active structural reorganization, such as the change of outer membrane permeability, the remodeling of intimal ridge structure, and the reorganization of membrane lipid distribution.

### 2.1 Mitochondrial Outer Membrane Permeability Triggers Apoptosis

**FIGURE 1:**
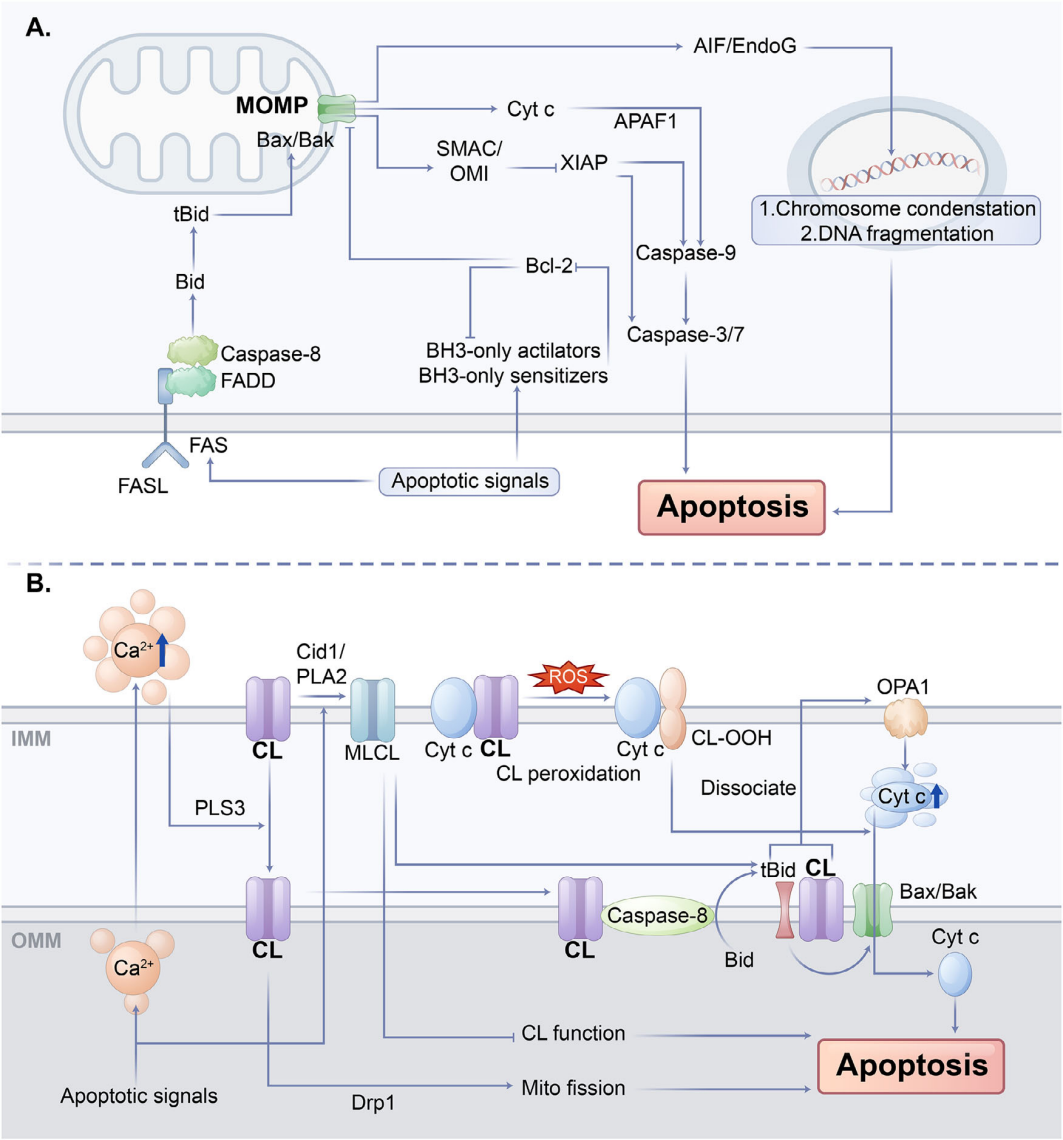
The role of mitochondrial membrane permeabilization and lipid distribution in apoptosis. (A) MOMP plays a crucial role in the mitochondrial‐dependent apoptotic pathway. Under the stimulation of apoptotic signals such as DNA damage, BH3‐only protein is activated and exhibits functional differentiation: the activating protein can directly stimulate the oligomerization of effectors Bax and Bak; and allergenic proteins can exert their effects by inhibiting antiapoptotic proteins. Activated Bax/Bak forms pore complexes in OMM, inducing MOMP and releasing intermembrane space proteins (Cyt *c*, SMAC, OMI, AIF, ENDOG), which exert proapoptotic effects through various pathways. (B) The translocation of CL from IMM to OMM is a key signal of apoptosis. CL can translocate by activating the Ca^2^^+^‐dependent PLS3 pathway. The proapoptotic protein Bid can also stimulate CL translocation. In promoting apoptosis, CL interacts with Drp1 and participates in mitochondrial fission. CL also acts on Bid, activating Bax/Bak to induce MOMP, thereby inducing apoptosis. The binding of Cyt *c* to CL helps anchor to IMM, and ROS peroxidation of CL facilitates the release of Cyt *c* into the cytoplasm. The CL–tBid complex can dissociate OPA1 oligomers, synergistically promote ridge remodeling, and induce apoptosis. In addition, under the stimulation of apoptosis, Cid1/PLA2 mediates the remodeling of CL acyl chains, transforming MLCL, which can lead to the loss of physiological ability of mature CL, and at the same time help (t)Bid relocate to mitochondria, thereby promoting Cyt *c* release and inducing apoptosis. MOMP, mitochondrial outer membrane permeation; Bcl‐2, B cell lymphoma‐2; BH3, Bcl‐2 homology domain 3; Bim, Bcl‐2‐interacting mediator of cell death; Bid, BH3‐interacting domain death agonist; PUMA, p53‐upregulated modulator of apoptosis; PMAIP1/NOXA, phorbol‐12‐myristate‐13‐acetate‐induced protein 1; Bad, Bcl‐2‐associated agonist of cell death; Bmf, Bcl‐2‐modifying factor; Bik, Bcl‐2‐interacting killer; Cyt *c*, cytochrome *c*; SMAC, second mitochondria‐derived activator of caspases; HtrA2/OMI, high‐temperature demand protein A2; AIF, apoptosis inducing factor; EndoG, endonuclease G; APAF1, apoptotic protease activator 1; XIAP, X‐linked inhibitor of apoptosis protein; CL, cardiac MLCL phospholipids; monodoluminergic phospholipids; ROS, reactive oxygen species; CL‐OOH, peroxidation of CL; OPA1, optic atrophy protein 1; Drp1, dynamin‐related protein 1.

Mitochondria, as a bilayer membrane organelle, are composed of the outer mitochondrial membrane (OMM) and the inner mitochondrial membrane (IMM), with the intermembrane gap between the two layers. The structural integrity of mitochondria plays a critical role in the cell. MOMP is the core of the mitochondrial‐dependent apoptotic pathway through Bcl‐2 family proteins (Figure 1A) [11, 12]. Under the stimulation of apoptotic signals such as DNA damage, BH3 only proteins are activated and exhibit functional differentiation: activating proteins such as Bim and Bid can directly stimulate the oligomerization of effectors Bax and Bak; stimulating proteins such as PUMA, PMAIP1 (also known as NOXA), Bad, Bmf, and Bik can exert their effects by inhibiting antiapoptotic proteins [13]. In addition, activated Bax/Bak forms pore complexes in OMM, induces MOMP, decreases membrane potential (ΔΨm), opens mitochondrial permeability transition pores (mPTP), increases permeability, and releases intermembrane space proteins (Cyt *c*, SMAC/DIABLO, HtrA2/OMI, AIF, and EndoG) [14]. Among them, Cyt *c* binds to APAF1 to form an apoptotic body, activating the Caspase‐9/Caspase‐3/7 cascade reaction to promote cell apoptosis [15]. SMAC/OMI binds to XIAP, blocking XIAP's inhibitory effect on caspase and promoting apoptosis [16, 17]. AIF and EndoG are translocated to the nucleus, causing chromosome condensation and DNA fragmentation in the nucleus, leading to cell apoptosis [18, 19]. In addition, in mouse spleen cells, Caspase‐8 of the FAS pathway can mediate Bid cleavage to generate tBid, synergistically activate the Bax/Bak–MOMP pathway, and form an apoptotic signal amplification loop [20].

### 2.2 Mitochondrial Lipid Redistribution Contributes to Apoptosis

OMM is rich in phosphatidylethanolamine and phosphatidylcholine, while IMM is mainly composed of cardiac phospholipids (CL), which also participate in the formation of mitochondrial respiratory chain complexes, mitochondrial dynamics regulation, and release of Cyt *c* [21]. The translocation of CL from IMM to OMM is a key signal of apoptosis (Figure 1B) [22]. After activation of death receptors, the Ca^2^^+^‐dependent PLS3 pathway is activated, promoting the translocation of CL from IMM to OMM [23]. The proapoptotic protein Bid can also stimulate CL translocation [24]. CL can also induce apoptosis by interacting with Drp1 and participating in mitochondrial fission [25]. In addition, CL can not only activate Caspase‐8 to cleave Bid into tBid, but also directly bind to tBid to promote its targeting of OMM. Subsequently, tBid induces MOMP by activating Bax/Bak, releasing Cyt *c* into the cytoplasm, and activating the caspase cascade reaction, thereby inducing apoptosis [26, 27]. The binding of Cyt *c* to CL helps anchor to IMM, and ROS peroxidation of CL leads to the release of Cyt *c* into the cytoplasm, which can serve as a signal to initiate cell apoptosis [28, 29]. And the CL–tBid complex can dissociate OPA1 oligomers, synergistically promote ridge remodeling, and induce apoptosis [30]. Research has found that under apoptotic stimulation, Cid1/PLA2 mediates the remodeling of CL acyl chains, transforming them into monodoluminergic phospholipids (MLCL) [31]. MLCL has different characteristics from CL, with poor stability in binding to proteins, leading to the loss of physiological ability of mature CL and participating in cell apoptosis [32]. The accumulation of MLCL helps tBid to be relocalized to mitochondria, thereby promoting Cyt *c* release and inducing apoptosis [33].

### 2.3 Crista Remodeling Induces Apoptosis

**FIGURE 2:**
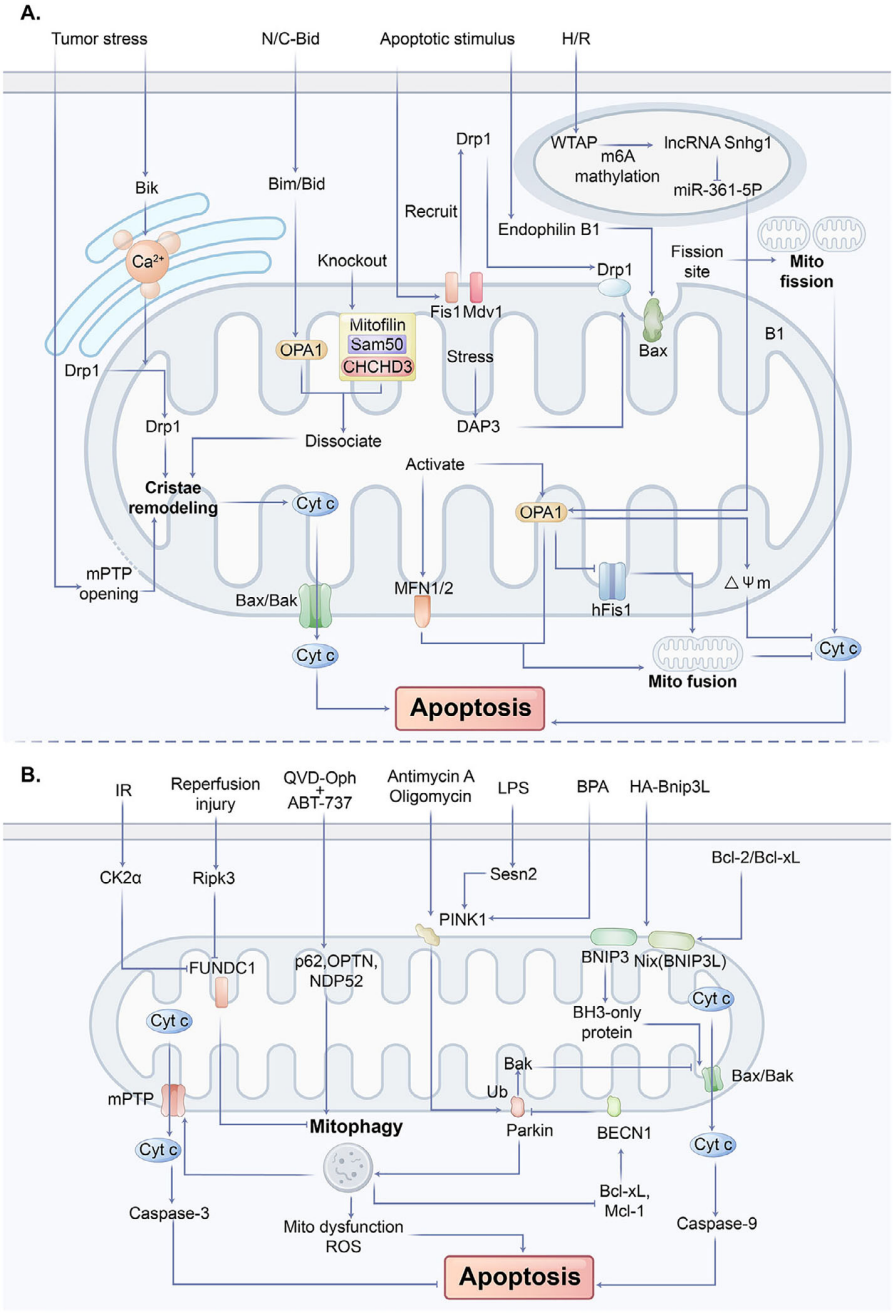
The role of crista remodeling and mitochondrial dynamic in apoptosis. (A) Cristae structure and mitochondrial fission and fusion in apoptosis.‌ MICOS (Mitoilin, Sam50, CHCHD3) and OPA1 maintain CJ integrity at the IMM. Mitoilin knockout disrupts cristae, increasing Cyt *c* release and ROS, promoting apoptosis. Under tumor stress, Bik activates Drp1 and mPTP, inducing cristae remodeling. Drp1 recruitment by Fis1/Mdv1p localizes with Bax, facilitating mitochondrial fission and Cyt *c* release before MOMP. DAP3 accelerates fission under stress, while Nefer B1 activates Bax/Bak. MFN2 inhibits Bax/Cyt *c* release. OPA1 inactivation reduces ΔΨm and triggers Cyt *c* release; WTAP promotes fusion via lncRNA Snhg1 m6A methylation, upregulating OPA1 and inhibiting hFis proapoptotic effects. (B) Mitochondrial autophagy and apoptosis crosstalk. Sesn2 activates PINK1/Parkin pathway to enhance mitophagy, reducing dysfunction and apoptosis in lipopolysaccharide‐induced inflammation. In contrast, Ripk3 inactivates FUNDC1 postreperfusion, impairing mitophagy and promoting Cyt *c* release. CK2α inhibition of FUNDC1 in cardiac IRI causes mPTP opening and Caspase‐3 activation. Bak ubiquitination via PINK1–Parkin inhibits its proapoptotic function. BPA disrupts energy metabolism via PINK1–Parkin, inducing apoptosis. Nix/BNIP3 release BH3 proteins to activate Bax/Bak, while Bcl‐xL/Mcl‐1 block Parkin translocation. ABT‐737 induces Bax/Bak‐dependent apoptosis, recruiting p62/OPTIN/NDP52 for organelle clearance via non‐PINK1 pathways. Bik, Bcl‐2‐interacting killer; Cyt *c*, cytochrome *c*; OPA1, optic atrophy protein 1; Drp1, dynamin‐related protein 1; MICOS, mitochondrial contact site and cristae organizing system; CJ, cristae junction; Sam50, sorting and assembly machinery 50; CHCHD3, oiled‐coil helix coiled‐coil helix domain‐containing protein 3; Fis1, fission 1 protein; DAP3, death‐associated protein 3; WTAP, RNA methyltransferase‐associated protein 1; MIRI, myocardial ischemia–reperfusion injury; MFN1/2, mitofusin 1/2.

A crista is a special structure formed by the inward folding of IMM, and its dynamic morphological changes are closely related to cell apoptosis by regulating the release of Cyt *c* (Figure 2A) [34]. The mitochondrial contact site and cristae organizing system complex and OPA1 are located at the cristae junction, responsible for IMM fusion and maintaining the integrity of the cristae junction [35]. Research has found that when isolated mouse liver mitochondria are incubated with recombinant N/C‐Bid, BH3 only proteins (such as Bim/Bid) can induce OPA1 oligomer depolymerization, leading to ridge junction opening, promoting Cyt *c* release, and triggering apoptosis [36]. In the mitochondrial contact site and cristae organizing system complex, Mitoilin is a core component that interacts with sorting and assembly machinery 50 (Sam50) and CHCHD3, responsible for repairing cristae structures [37]. Knocking out *Mitoilin* in H9C2 cardiomyocytes and human embryonic kidney cells leads to disruption of cristae structure, increased release of Cyt *c*, and accumulation of reactive oxygen species (ROS), thereby promoting apoptosis [38]. In addition, under the stimulation of tumor stress, Bik promotes the release of calcium ion (Ca^2+^) from the ER and Drp1 to mitochondria. At the same time, the opening of mPTP further synergizes with Drp1 to induce cristae remodeling and accelerate the process of apoptosis [39].

In conclusion, mitochondria are not only the “energy factory” of cells, and the structural and functional integrity of their outer membrane, inner membrane (cristae), and membrane lipids together constitute a precise apoptotic regulatory network. The dynamic changes of these three layers are interrelated and synergetic, which together determine the fate of cell apoptosis after receiving the death signal.

## 3 Mitochondrial Dynamic Imbalance Contributes to Apoptosis

Mitochondria are not static organelles, and they maintain their own quality and functional homeostasis through continuous fusion and division (mitochondrial dynamics) and selective autophagy. The destruction of this dynamic balance is the key mechanism to promote the process of cell apoptosis [6, 40]. Mitochondria are in a dynamic equilibrium state within cells, maintaining functional homeostasis through continuous fusion and fission processes, which is known as mitochondrial dynamics. Fusion helps mix mitochondrial contents and maintain functional integrity, while fission enables mitochondria to adapt to changing cellular energy demands (Figure 2A). When cells are damaged, defective mitochondria are removed through the autophagy pathway [41].

### 3.1 Mitochondrial Fission Promotes Apoptosis

Mitochondrial fission requires the involvement of proteins such as Drp1, Fis1, endostatin B1/Bif‐1, DAP3, and *Fis2* gene product (called Mdv1). Mitochondrial fission itself usually does not cause cell apoptosis, but rather plays a “sensitization” role [41]. During the process of cell apoptosis, Fis1 and Mdv1 can promote the recruitment of Dnm1p (the yeast homolog of Drp1) to mitochondria [41, 42]. Drp1 forms lesions at the fracture site and tip, colocalizes with the proapoptotic protein Bax, and induces apoptosis downstream of Bax translocation and upstream of Cyt *c* release [43, 44]. Meanwhile, Drp1 stably binds to the mitochondrial membrane in a Bax/Bak‐dependent manner, facilitating the remodeling of the mitochondrial inner and outer membranes and cristae before the complete release of MOMP and Cyt *c* [45]. DAP3 is a mitochondrial ribosome‐associated matrix protein, and overexpression under stress conditions can specifically promote fission and accelerate Cyt *c* release [46]. Neipro B1 promotes apoptosis by enhancing the recruitment and activation of Bax/Bak [47].

### 3.2 Reduced Mitochondrial Fusion Induces Apoptosis

During apoptosis, mitochondrial fusion is reduced, MFN1/2 (located in OMM) and OPA1 (located in IMM). Its dysfunction is closely related to apoptosis: knocking down mammalian *MFN1/2* and *OPA1* increases mitochondrial fragmentation and sensitivity to apoptosis [44, 48]. The main active form of MFN2 can inhibit Bax activation and Cyt *c* release and prevent free radical‐induced permeability transition, thereby inhibiting cell apoptosis [49]. The apoptosis induced by OPA1 inactivation is accompanied by a decrease in ΔΨm and release of Cyt *c*, which can be reversed by Bcl‐2 [50]. Studies have found that RNA methyltransferase WTAP can induce m6A methylation of lncRNA Snhg1, thereby inhibiting microRNA‐361‐5p and upregulating OPA1 to promote mitochondrial fusion in mouse cardiomyocytes, alleviating myocardial ischemia–reperfusion (I/R) injury (IRI) [51]. In addition, OPA1 may play a role in inhibiting the apoptosis‐promoting function of human Fis [44].

### 3.3 The Dual Role of Mitochondrial Autophagy in Apoptosis

Mitochondrial autophagy has a dual regulatory effect on cell apoptosis: under reversible injury conditions, mitochondrial autophagy clears damaged mitochondria to inhibit apoptosis. However, some excessive stimulation will abnormally activate mitochondrial autophagy, cause changes in energy metabolism, and promote apoptosis (Figure 2B). This dual effect depends to some extent on the stress intensity, the duration of injury, and the functional differences of specific autophagy receptors. Under mild stress, early disease, or reversible injury, mitochondrial autophagy can selectively clear dysfunctional mitochondria, thereby reducing the potential death signals contained in cells and inhibiting apoptosis. Research has found that in the lipopolysaccharide‐induced mouse macrophage inflammation model, upregulation of Sesn2 enhances mitochondrial autophagy by activating the PINK1/E3 ubiquitin ligase (Parkin) pathway, thereby attenuating mitochondrial dysfunction and ROS levels, then reducing cell apoptosis and delaying the progression of severe acute pancreatitis [52]. Similarly, in the initial stage of heart failure, if the reduced level of mitochondrial autophagy is appropriately upregulated, the disease progression can be delayed [53]. In myocardial reperfusion injury, appropriately increasing mitochondrial autophagy can reduce the accumulation of dysregulated mitochondria and play a protective role [54, 55]. However, under severe/persistent stress, advanced disease, or irreversible damage, excessive mitochondrial autophagy will promote apoptosis, thereby aggravating the damage. In the late stage of heart failure, mitochondrial autophagy is abnormally activated, which destroys the function of myocardial cells, promotes cell apoptosis and aggravates symptoms [56]. Similarly, in myocardial reperfusion injury, it is also found that excessive stimulation of mitochondrial autophagy will lead to energy crisis, which will increase the burden [54, 55]. Thus, maintaining an appropriate level of mitochondrial autophagy is conducive to disease control and treatment. In addition, the bidirectional regulation of mitochondrial autophagy on apoptosis may be related to the functional differences of autophagy receptors. For example, autophagy receptor FUNDC1 plays a protective role in inhibiting apoptosis by recognizing damaged mitochondria and promoting autophagy. During ischemia, the activated FUNDC1 promotes mitochondrial autophagy, reduces Cyt *c* leakage into the cytoplasm, and inhibits Caspase‐9‐related cell apoptosis; but after reperfusion, upregulation of Ripk3 leads to the inactivation of FUNDC1, resulting in decreased autophagy ability, inducing Caspase‐9‐mediated cell apoptosis [57]. In a mouse model of cardiac IRI, upregulation of CK2α inhibits FUNDC1‐mediated autophagy, leading to mPTP opening and Cyt *c* leakage, activating Caspase‐3‐dependent apoptosis; knocking out *CK2α* can reverse this process and have a protective effect on the heart [58]. In contrast, Bcl‐2 and BNIP3 and other receptors are also proapoptotic proteins, which have the dual potential of promoting autophagy and apoptosis. The change of their functions depends on the stress intensity to some extent. Under moderate stress, it mainly plays the role of autophagy receptor, scavenging damaged mitochondria and inhibiting apoptosis, playing a protective role. Some studies have established myocardial IRI rat model and found that HIF‐1α/BNIP3 pathway is activated, which then promotes mitochondrial autophagy, and can effectively reduce the apoptosis of H9C2 cardiomyocytes in rats [59]. However, under the condition of excessive stress, excessive autophagy mediated by the same receptor or direct proapoptotic effect may lead to damage. In acute cardiac IRI, DUSP1 in mouse cardiomyocytes is downregulated, which activates the activation of JNK, thus overactivating BNIP3‐mediated mitochondrial autophagy, significantly consuming mitochondrial mass, leading to mitochondrial metabolic disorders, and promoting cell apoptosis to aggravate the injury [60]. It was also found that BNIP3 was significantly upregulated by hypoxia/reoxygenation treatment of H9C2 cells to simulate myocardial IRI and overactivated mitochondrial autophagy‐induced cell apoptosis [61]. In particular, the high expression of BNIP3 sometimes helps the metabolic reprogramming of tumor cells and promotes tumor invasion and metastasis [56]. Based on the complexity and dynamics of mitochondrial autophagy regulating apoptosis, further research is needed to clarify the specific molecular mechanism of mitochondrial autophagy dual regulating apoptosis and systematically clarify the interaction and core regulatory network in the spatio‐temporal dynamic development of disease. In addition, it is necessary to define the threshold of mitochondrial autophagy from protection to destruction in terms of stimulation intensity, time dynamics of injury, and functional characteristics of specific autophagy receptors, so as to dynamically regulate the balance of autophagy and apoptosis in the process of disease development, so as to provide treatment strategies for delaying or even reversing the development of disease.

Second, mitochondrial autophagy can also regulate cell apoptosis by affecting proteins that regulate apoptosis. After mitochondrial damage, Bak can be ubiquitinated through the classical PINK1–Parkin pathway, inhibiting Bak oligomerization and its apoptotic function of releasing Cyt *c* from intermembrane gap. This ensures efficient clearance of damaged mitochondria by autophagosomes while avoiding abnormal activation of apoptotic signals [62]. But when excessively damaged, the PINK1–Parkin pathway can also induce apoptosis [63]. Research has shown that environmental doses of bisphenol A can induce energy metabolism imbalance in hippocampal neurons through PINK1/Parkin‐mediated mitochondrial autophagy. This process upregulates proapoptotic proteins such as Cyt *c*, Bax, Bak1, and Caspase‐3, while downregulating the antiapoptotic protein Bcl‐2, thereby inducing apoptosis. These findings provide a basis for understanding the mechanisms of mitochondrial dysfunction in neurological diseases [64]. In addition, studies have shown that receptors mediating mitochondrial autophagy, such as BNIP3 and BNIP3‐like (BNIP3L, also known as Nix), can directly activate apoptosis. After transient transfection of Rat‐1 and HeLa cells with recombinant HA‐BNIP3L expression plasmid or empty vector, Nix and BNIP3 bind to Bcl‐2/Bcl‐xL through the carboxyl terminal domain, releasing BH3 only protein to activate Bax/Bak, induce MOMP and Cyt *c* release, and promote apoptosis [65].

Some proteins that regulate apoptosis can also affect the progress of mitochondrial autophagy. The Bcl‐2 family antiapoptotic proteins (Bcl‐xL and Mcl‐1) can block Parkin translocation to mitochondria and inhibit mitochondrial autophagy by binding to beclin‐1 [66]. The latest research shows that BH3 mimic ABT‐737 induces Bax/Bak‐dependent apoptosis in mouse embryonic fibroblasts, and after treatment with the pan caspase inhibitor QVD‐Oph, mitochondrial damage during apoptosis leads to IMM exposure, triggering IMM ubiquitination and recruiting autophagy adaptor proteins p62, OPTN, and NDP52, bypassing the classical PINK1/Parkin and STING pathways, initiating mitochondrial autophagy through Bak/Bax‐dependent mechanisms, targeting damaged mitochondria for lysosomal degradation, and achieving organelle clearance [67].

In conclusion, the accelerated mitotic division, the blocked fusion, and the imbalance of autophagy constitute a fine dynamic regulatory network. They play an important role in the choice of cell fate by directly affecting the integrity of mitochondrial membrane, the release of proapoptotic factors, and the clearance efficiency of damaged organelles.

## 4 The Release, Damage, and Mutation of mtDNA Induce Apoptosis

**FIGURE 3:**
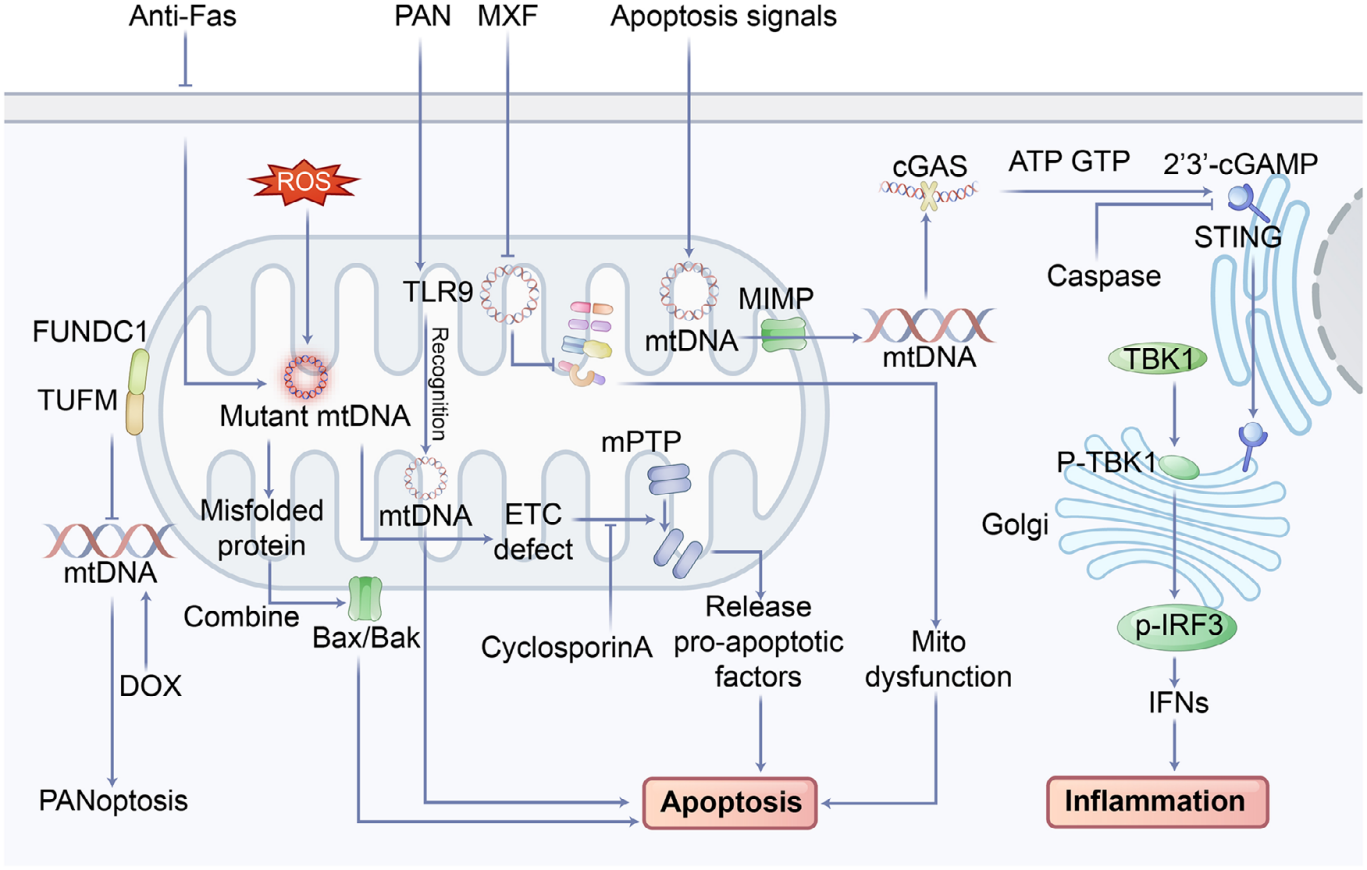
The role of mitochondrial DNA in apoptosis. MtDNA cytoplasmic mtDNA activates the TBK1–IRF3 axis through the cGAS/STING pathway, inducing IFNs production, amplifying inflammatory responses, and leading to cell death; MXF can damage mtDNA, causing severe dysfunction of Complexes I, III, IV, and ATP synthase, leading to mitochondrial dysfunction and ultimately inducing cell apoptosis; under oxidative stress such as ROS or replication errors, mtDNA undergoes mutations, leading to defects in the electron transfer chain, and abnormal opening of mPTP, releasing proapoptotic factors and triggering cell apoptosis; cyclosporine A can inhibit mPTP opening; by treating leber hereditary optic neuropathy cells with anti‐Fas, it was found that mtDNA mutations disrupt the regulatory effect of Complex I on mPTP and opening it up, thereby increasing the sensitivity of cells to Fas apoptotic signals; mtDNA mutations can produce a set of misfolded mitochondrial proteins, which may have the conformation required to bind to Bax or Bak, thereby activating cell apoptosis; TLR9 promotes podocyte apoptosis by recognizing endogenous mtDNA; DOX can induce cytoplasmic mtDNA release triggers PANoptosis, while autophagy receptor FUNDC1–TUFM inhibit PANoptosis. PINK1, phosphatase and tensin homolog‐induced kinase 1; Parkin, E3 ubiquitin ligase; SAP, severe acute pancreatitis; FUNDC1, FUN14 domain containing 1; Ripk3, receptor‐interacting protein kinase 3; MXF, moxifloxacin; BNIP3, Bcl‐2 and adenovirus E1B 19 kDa‐interacting protein 3; BNIP3L, BNIP3‐like; BPA, bisphenol A; Bcl‐xL, B‐cell lymphoma‐extra‐large; Mcl‐1, myeloid cell leukemia‐1; OPTN, optineurin; NDP52, nuclear dot protein 52; TBK1, TANK‐binding kinase‐1; IRF3, interferon regulatory factor 3; cGAS, cyclic GMP–AMP synthase; STING, stimulator of interferon genes; IFN, interferons; MXF, moxifloxacin; ETC, electron transfer chain; PAN, puromycin aminonucleosid; TLR9, Toll‐like receptor 9; DOX, doxorubicin; TUFM, Tu translation elongation factor.

MtDNA is not only the core genetic material for mitochondrial energy metabolism but also participates in apoptosis regulation as a damage signaling molecule (Figure 3). Its mechanism of action includes both proinflammatory response and direct promotion of apoptosis [16]. When cells undergo apoptosis, mtDNA can be released to participate in proinflammatory responses, and it is an effective damage‐related signaling molecule that triggers innate immunity when it reaches the cytoplasm or extracellular environment [68]. However, the release of mtDNA is driven by oxidative damage, membrane permeability, and a variety of cell death pathways [69, 70]. In apoptotic mouse fibroblasts, activation of Bak/Bax and release of Cyt *c* lead to the formation of MOMP, accompanied by mitochondrial inner membrane permeabilization, and then mtDNA is released into the cytoplasm through inner membrane vesication [71], which is independent of mitochondrial dynamics and permeability transition [72]. It is worth noting that VDAC is the most abundant protein in OMM [73]. Under oxidative stress and other mitochondrial stress conditions, VDAC can realize oligomerization, and then form large OMM pores, which can release mtDNA into the cytoplasm [73]. Ca^2+^ in the ER can be transferred to mitochondria through IP3R, and when Ca^2+^ is overloaded, mPTP is activated and VDAC oligomerization is triggered, resulting in the release of mtDNA [74]. VADC and IP3R are important components of mitochondrial‐associated ER membranes (MAMs), so how MAMs affect mtDNA release remains to be further explored. Cytoplasmic mtDNA activates the TBK1–IRF3 axis through the DNA‐sensing enzyme cGAS/STING, inducing IFN production, amplifying inflammatory responses, and leading to cell death [74, 75]. The cascade reaction of caspases, although unable to prevent the formation of inner membrane vesicles, can inhibit mtDNA‐mediated intrinsic immune signal transduction by disrupting dead cells [71].

MtDNA is also involved in the process of cell apoptosis (Figure 3). On the one hand, mtDNA is involved in encoding important components of the mitochondrial respiratory chain, regulating oxidative phosphorylation, so if mtDNA is damaged, it can lead to impaired energy metabolism and oxidative stress, ultimately inducing apoptosis [76]. The use of antibiotic moxifloxacin to treat NK cells can damage mtDNA, causing severe dysfunction of Complexes I, III, IV, and adenosinetriphosphate (ATP) synthase, leading to mitochondrial dysfunction and ultimately inducing cell apoptosis, with immunomodulatory effects [76]. On the other hand, under oxidative stress, such as ROS or replication errors, mtDNA undergoes mutations, leading to defects in the electron transfer chain and abnormal opening of mPTP, releasing proapoptotic factors and triggering cell apoptosis [77]. In a mouse model with heart‐specific mitochondrial mutations, cyclosporine A prevents mtDNA mutation‐related cardiac damage by inhibiting the opening of the mPTP [78]. Leber hereditary optic neuropathy is caused by mtDNA mutations. By treating leber hereditary optic neuropathy cells with anti‐Fas, it was found that mtDNA mutations affect the encoding genes of Complex I subunits in the mitochondrial electron transport chain, disrupting the regulatory effect of Complex I on mPTP and opening it up, thereby increasing the sensitivity of cells to Fas apoptotic signals [79, 80]. At the same time, mtDNA mutations can produce a set of misfolded mitochondrial proteins, a small portion of which may have the conformation required to bind to Bax or Bak, thereby activating cell apoptosis [81].

In addition, in the puromycin aminonucleoside‐induced rat nephrotic syndrome model, TLR9 promotes podocyte apoptosis by recognizing endogenous mtDNA, which is a novel mechanism of podocyte injury in glomerular diseases [82]. The latest research has revealed a programmed cell death pattern called PANoptosis, which is induced by the synergistic effects of pyroptosis, apoptosis, and necroptosis [83]. In a mouse model of myocardial injury induced by doxorubicin (DOX), cytoplasmic mtDNA release triggers pan apoptosis, while autophagy receptor FUNDC1 reduces cytoplasmic release of mtDNA through interaction with TUFM, inhibiting pan apoptosis of myocardium [84].

## 5 Mitochondria Crosstalk With Other Organelles Contributes to Apoptosis

Mitochondria are not only the “energy factory” of cells, but also the central hub for integrating metabolism, stress, and survival signals. It forms a highly dynamic intracellular signal network by establishing close physical and functional connections with organelles such as ER, lipid droplets (LDs), nucleus, and lysosome and jointly regulates energy homeostasis, calcium signaling, lipid metabolism, autophagy, and cell fate.

### 5.1 Mitochondria Crosstalk With the ER Contributes to Apoptosis

**FIGURE 4:**
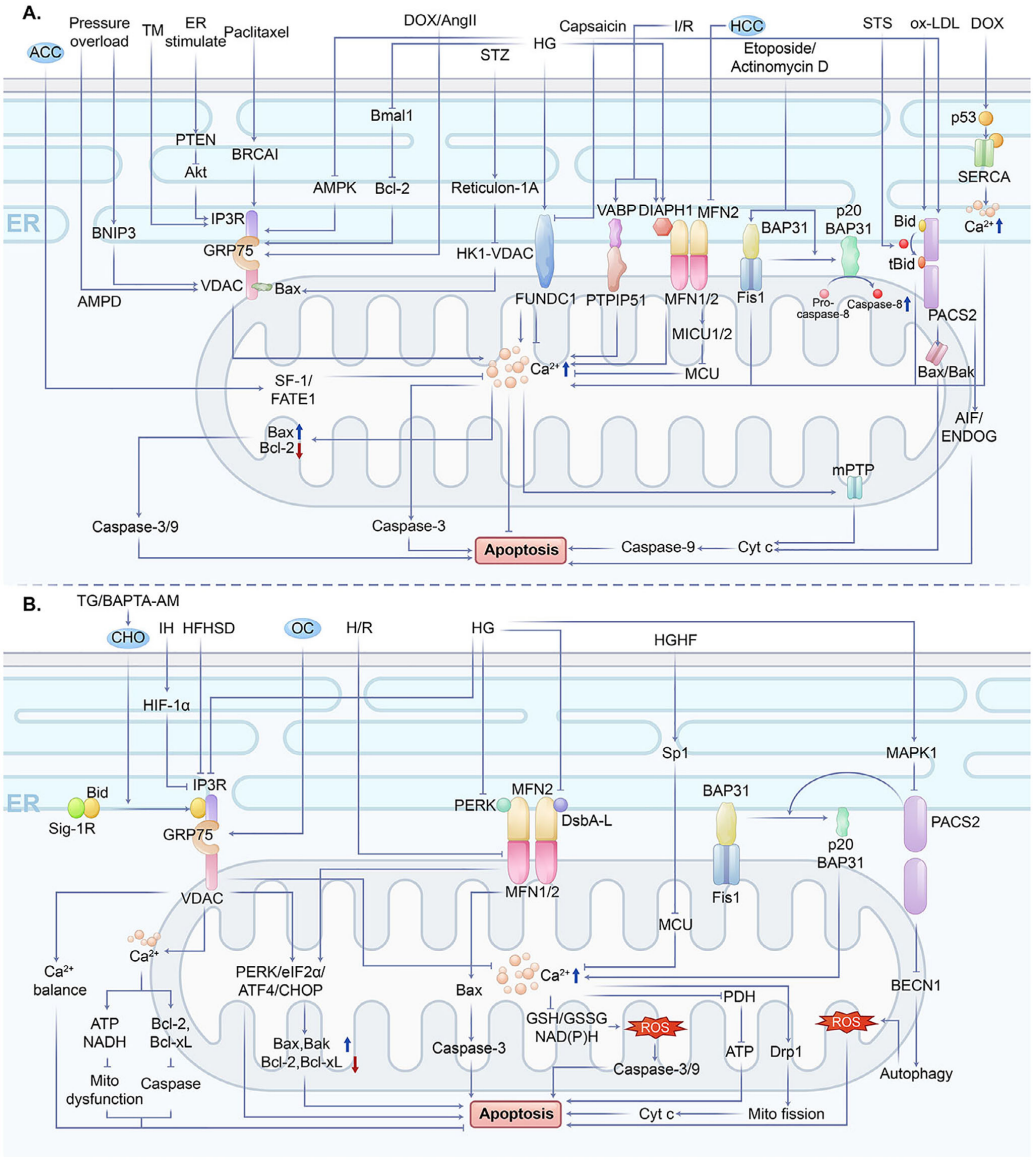
The role of mitochondria–ER interaction in apoptosis of disease and cancer. Mitochondria‐associated membranes (MAMs) regulate Ca^2^^+^ signaling and apoptosis via protein complexes. (A) MAM enhancement promotes apoptosis high glucose (HG) upregulates MFN2, PACS2, and FUNDC1, increasing MAM formation and mitochondrial Ca^2^^+^ influx while inhibiting AMPK and Bcl‐2. Pressure overload, doxorubicin/Ang II, and tunicamycin similarly enhance MAMs, leading to Ca^2^^+^ overload and apoptosis. Capsaicin inhibits MAMs via TRPV1–AMPK, reducing Ca^2^^+^ uptake. For MAM and cancer, DOX activates p53, enhancing SERCA activity and mitochondrial Ca^2^^+^ overload. PTX upregulates BRCA1, increasing IP3R1 sensitivity to Ca^2^^+^ and promoting apoptosis. (B) MAM disruption induces apoptosis HG downregulates MFN2, dissociating MAM complexes and activating PERK/eIF2α/ATF4/CHOP. MAPK1 downregulates PACS2, causing mtROS accumulation and Bax/Caspase‐3 activation. High‐fat, high‐sucrose diet and high glucose and high‐fat disrupt Ca^2^^+^ channels, while hypoxia and IRI impair MAM integrity, triggering Ca^2^^+^ dysregulation and apoptosis. And HCC downregulates MFN2, disrupting MAM and upregulating MICU1/2 to avoid apoptosis. ACC cells reduce MAM via SF‐1/FATE1, evading apoptosis. FUNDC1, FUN14 domain containing 1; MFN1/2, mitofusin 1/2; ERS, endoplasmic reticulum stress; ER, endoplasmic reticulum; MAM, mitochondrial‐associated ER membrane; IP3R, inositol 1,4,5‐triphosphate receptor; GRP75, glucose regulatory protein 75 kDa; VDAC, voltage‐dependent anion channel; MCU, mitochondrial calcium uniporter;VAPB, vesicle‐associated membrane protein B; PTPIP51, protein tyrosine phosphatase interaction protein 51; SERCA, sarcoplasmic/endoplasmic reticulum Ca^2+^‐ATPase; BAP31, B cell receptor‐associated protein 31; PACS2, phosphofuran acidic cluster sorting protein 2; Bmal1, brain and muscle ARNT‐like protein‐1; AMPK, AMP activated protein kinase; AMPD, adenosine monophosphate deaminase; HK, hexokinase; TRPV1, transient receptor potential vanilloid 1; DIAPH1, diaphanous1; POH, pressure‐overload hypertrophy; PERK, pancreatic ER kinase‐like ER kinase; eIF2α, eukaryotic initiation factor 2α; ATF4, ativating transcriptional factor 4; CHOP, C/EBP homologous protein; DsbA‐L, disulfide‐bond A oxidoreductase‐like protein; MAPK1, mitogen activated protein kinase1; IH, intermittent hypoxia; HIF‐1α, hypoxia inducible 2 factor‐1α; IRI, ischemia–reperfusion injury; BRCA1, breast cancer 1 protein; PTEN, phosphatase and tensin homolog; Akt, protein kinase B; MICU, mitochondrial Ca^2+^ uptake; SF‐1, steroidogenic factor‐1; HCC, hepatocellular carcinoma; TG, thapsigargin; BAPTA‐AM, BAPTA acetoxymethyl ester; Sig‐1R, sigma‐1 receptor; BiP, binding immunoglobulin protein; FATE1, fetal and adult testis expressed 1; ACC, adrenal cortical cancer; OC, ovarian cancer; CHO, Chinese hamster ovary.

MAMs are membrane components that are coupled between mitochondria and ER, which mainly affects the level of apoptosis by regulating Ca^2+^ through MAM structure, and its destruction and promotion will promote apoptosis through multiprotein complexes (Figure 4) [85]. The GRP75–VDAC complex forms a Ca^2+^ regulatory axis with MCU, MFN2–MFN1/2, and ER membrane protein VAPB–OMM PTPIP51 complex mediate Ca^2+^ transfer from ER to mitochondria, causing mitochondrial Ca^2+^ overload, inducing mPTP opening, and releasing proapoptotic factors such as Cyt *c* [86]. At the same time, RyR at the ER releases intracellular Ca^2+^, while the SERCA recovers cytoplasmic Ca^2+^, jointly maintaining calcium homeostasis [10]. In addition, BAP31, located in the ER, forms a complex with Fis1, participating in another pathway of MAM‐induced apoptosis, while PACS2 regulates mitochondrial binding to ER in a BAP31‐dependent manner [10].

#### 5.1.1 Enhancing MAM Induces Apoptosis to Accelerate the Progression of Diseases

Enhancing MAM induces apoptosis mainly by inducing calcium overload to accelerate the progression of diseases (Figure 4A). Most studies have found that high glucose can accelerate the progression of diabetes‐related diseases by enhancing the formation of MAM, inducing apoptosis. HG can stimulate primary atrial myocytes in rats to accelerate ER stress (ERS) and upregulate MFN2, shorten the distance between ER and mitochondria, thereby increasing the uptake of Ca^2+^ by atrial myocyte mitochondria and inducing cardiomyocyte apoptosis [87]. In rat cardiomyocytes, HG upregulates PACS2, IP3R2, VDAC1, and FUNDC1, increasing the formation of MAM and thereby upregulating the mitochondrial‐driven AIF/ENDOG–caspase pathway [88]; At the same time, HG downregulates brain and muscle Bmal1, reduces Bcl‐2 expression, weakens its inhibitory effect on IP3R, promotes the formation of MAM, leads to IP3R–VDAC1‐mediated MAM‐related Ca^2+^ overload and ROS accumulation, triggers mPTP opening, releases Cyt *c* and SMAC, activates Caspase‐9/3 cascade leading to cardiomyocyte apoptosis, and promotes the progression of diabetes cardiomyopathy [89]. In addition, in a Type 1 diabetic mouse model, HG inhibits the activity of AMPK in cardiomyocytes and upregulates the autophagy receptor FUNDC1. FUNDC1 binds to and stabilizes IP3R2, thereby enhancing mitochondria‐associated membrane (MAM)‐mediated mitochondrial Ca^2^^+^ influx, inducing prolonged opening of the mPTP, promoting cardiomyocyte apoptosis, and ultimately reducing myocardial contractility [90]. The latest research shows that in the model of Type 2 diabetes rats, pressure overload upregulates AMPD, enhances the formation of MAMs through VDAC interaction, leads to Ca^2+^ overload and cardiomyocyte apoptosis, leads to diastolic function, and aggravates diabetes cardiomyopathy [91].

In addition, in the DOX/Angiotensin II mouse podocyte model, upregulation of IP3R1–GRP75–VDAC1 complex and MCU expression induced mitochondrial Ca^2+^ overload, activated Caspase‐3‐dependent podocyte apoptosis, and accelerated the progression of refractory nephrotic syndrome [92]. In the streptozotocin/OVE26 diabetes mouse model, the overexpression of endoplasmic reticulon‐1A aggravates ERS, inhibits HK1–VDAC interaction, promotes Bax/VDAC oligomerization and Cyt *c* release, leads to apoptosis of renal tubular cells, and worsens diabetic kidney disease [93]. At the same time, in the podocyte model of db/db diabetes mice, capsaicin activates the transient receptor potential vanilloid 1–AMPK pathway, inhibits the formation of MAMs mediated by FUNDC1, reduces mitochondrial Ca^2+^ uptake, and reduces the occurrence of diabetic kidney disease, suggesting a potential therapeutic target [94].

MAM is also a therapeutic target for cardiovascular diseases. The accumulated oxidized low‐density lipoprotein‐treated human umbilical vein endothelial cells enhanced MAMs by upregulating PACS2, triggered the imbalance of ER mitochondrial Ca^2+^ cycle, Ca^2+^ overload led to the accumulation of ROS, triggered the opening of mPTP and the release of Cyt *c*, caused Caspase‐9‐mediated endothelial cell apoptosis, and promoted atherosclerosis [95]. IRI can act through MAM. In the I/R mouse model, upregulation of PTPIP51 and enhancement of DIAPH1–MFN2 interaction promote MAMs‐dependent Ca^2+^ overload, trigger mPTP opening, and lead to cardiomyocyte apoptosis [96, 97, 98]. In the ERS model, tunicamycin treatment induced mitochondrial Ca^2+^ overload in mouse atrial myocytes through IP3R1–GRP75–VDAC1 complex, downregulated Bcl‐2, and upregulated Bax, activated Caspase‐3/9, and drove atrial remodeling and progression of atrial fibrillation [99]. In the pressure‐overload hypertrophy rat model, upregulation of BNIP3 promotes VDAC oligomerization, enhances MAMs‐mediated Ca^2+^ influx, triggers cardiomyocyte apoptosis, and heart failure progression [100].

It is worth noting that the enhancement of MAM can upregulate the sensitivity of cancer cells to apoptotic signals, and may become a new target for cancer therapy. Studies have shown that treatment with DOX in human non‐small cell lung, cervical, and breast cancer cells activates p53, causing its accumulation in MAMs. p53 then interacts with the SERCA pump to enhance its activity, leading to mitochondrial Ca^2^^+^ overload and subsequently inducing apoptosis in cancer cells [101]. In HeLa cells of human cervical cancer, paclitaxel (PTX) regulates IP3R1 activity by upregulating BRCA1, which is recruited to the ER surface in an IP3R‐dependent manner. By enhancing IP3R1's sensitivity to IP3, PTX promotes Ca^2+^ transport to mitochondria and initiates apoptosis [102]. High concentrations of staurosporin can drive the translocation of PACS2–full‐length Bid complex from ER to mitochondria in melanoma cells. Caspase‐8 cleaves Bid on OMM, and its active fragment promotes Cyt *c* release through Bax/Bak‐mediated MOMP, ultimately activating the Caspase‐3 cascade reaction and initiating cell apoptosis [103]. In addition, treatment with etoposide or actinomycin D can induce the binding of Fis1 to BAP31, which undergoes protein hydrolysis to generate the proapoptotic protein p20 BAP31. This protein has a dual function: it can activate pro‐Caspase‐8 to initiate apoptotic signals and promote Ca^2+^ transfer between ER mitochondria to enhance apoptotic effects [104]. In addition, under the stimulation of ER death, the classic tumor suppressor PTEN is upregulated, which enhances the stability of MAM by inhibiting PKB (also known as AKT)‐mediated IP3R3 phosphorylation, thereby promoting Ca^2+^ transfer to mitochondria and inducing apoptosis. This discovery provides a new perspective for understanding the tumor suppressive function of PTEN [105].

In addition, cancer cells also evade apoptosis through MAM. The downregulation of MFN2 protein expression in hepatocellular carcinoma tissue disrupts MAMs and subsequently upregulates the mitochondrial Ca^2^^+^ uptake “gatekeepers” MICU1 and MICU2. This maintains the MCU in a closed state, inhibits Ca^2^^+^ transfer from the ER to mitochondria, prevents hepatocyte apoptosis, and is associated with poor prognosis [106]. Activation of the SF‐1/FATE1 axis in adrenal cortical cancer cells can significantly reduce the contact area between ER and mitochondria, decrease Ca^2+^ metastasis, and inhibit Caspase‐3/7 activity, thereby resisting the action of chemotherapy drug mitoxan by evading apoptosis [107]. It can be seen that some cancers may escape apoptosis by downregulating MAM and reversibly turn to drug resistance against this mechanism.

#### 5.1.2 Disrupting MAM Integrity Promotes Apoptosis

The dynamic balance of MAM is crucial for cell survival. Damage to its integrity can lead to calcium homeostasis imbalance, mitochondrial dysfunction, and abnormal activation of apoptotic signals (Figure 4B). Therefore, the decrease in MAM integrity can lead to cell apoptosis [108]. In diabetes nephropathy, the destruction of MAM will lead to apoptosis in most cases. HG stimulates human podocytes to downregulate MFN2 expression, dissociate MFN2–PERK, and disrupt MAM, thereby activating the PERK/eIF2α/ATF4/CHOP pathway, upregulating proapoptotic proteins Bax and Bak, and inhibiting antiapoptotic proteins Bcl‐2 and Bcl‐xL, thereby inducing podocyte apoptosis [109]. In HG‐treated human proximal tubular epithelial cells and streptozotocin‐induced diabetic mouse models, inhibition of the DsbA‐L/MFN2 axis leads to loss of MAM integrity, increased mitochondrial Bax and Caspase‐3 expression, and promotes renal tubular apoptosis and kidney injury [108]. Moreover, upregulation of MAPK1 decreases PACS2 expression, on the one hand, the downregulation of PACS2 can induce the cleavage of BAP31 into proapoptotic fragment BAP31 P20, leading to ER mitochondrial Ca^2+^ overload, and then Drp1‐mediated mitochondrial disruption and Cyt *c* release; on the other hand, it can also block its binding with BECN1, leading to mitochondrial clearance disorder and mitochondrial ROS accumulation, aggravating apoptosis [110, 111]. In addition, in the diabetes kidney models of Type 1 and Type 2 mice, the interaction between IP3R1–VDAC1 and Grp75–VDAC1 was reduced, which decreased the MAM contact, stimulated the PERK/eIF2α/ATF4/CHOP pathway, and induced apoptosis of renal tubular cells [112].

Specifically, in a Type 2 diabetes mouse model induced by a high‐fat, high‐sucrose diet, the formation of the IP3R1/Grp75/VDAC1 Ca^2^^+^ channel complex in cardiomyocytes is reduced. This leads to decreased mitochondrial Ca^2^^+^ uptake, inhibition of pyruvate dehydrogenase activity, and a decline in ATP synthesis, resulting in myocardial contractile dysfunction and the early onset of diabetes cardiomyopathy [113]. High glucose and high‐fat treatment of rat cardiomyocytes upregulates Sp1, inhibits the formation of MCU complexes, reduces mitochondrial Ca^2^^+^ uptake, decreases mitochondrial dehydrogenase activity, and disrupts the balance of glutathione/glutathione disulfide (GSH/GSSG) and nicotinamide adenine dinucleotide (phosphate). This leads to the accumulation of mitochondrial ROS, activation of Caspase‐9 and Caspase‐3, and ultimately induces cardiomyocyte apoptosis [114]. During intermittent hypoxia, hypoxia HIF‐1α is upregulated, leading to a decrease in VDAC/IP3R1 interaction, an imbalance in Ca^2+^ homeostasis, and induction of cardiomyocyte apoptosis [115]. In addition, in the hypoxia/reoxygenation‐induced rat IRI model, MFN2 is downregulated, MAM is disrupted, leading to Ca^2+^ disorder, ERS, and enhanced mitochondrial dysfunction, inducing cell apoptosis, and participating in the development of acute kidney injury [116, 117].

Different from the previously mentioned theory, enhancing MAM can cause the drug resistance of some cancer cells (Figure 4B). This may be because the excessive enhancement of MAM can help tumor adapt to stress by enhancing mitochondrial metabolism, thus avoiding apoptosis. In the formation of MAM, the enrichment of GRP75–VDAC1 complex in human ovarian cancer cells is closely related to cisplatin (CP or CDDP) resistance. Knocking out *GRP75* can decrease MAM integrity and overcome drug resistance through a dual mechanism. First, it reduces the transfer of Ca^2^^+^ from the ER to mitochondria, leading to decreased ATP and NADH levels in ovarian cancer cells, thereby accelerating cisplatin‐induced mitochondrial dysfunction. Second, it disrupts the colocalization of GRP75 with Bcl‐2/Bcl‐xL, enhancing caspase‐dependent apoptosis in OC cells. Thus, GRP75 is a potential target for overcoming drug resistance [118]. Under the stimulation of thapsigargin and calcium chelator BAPTA acetoxymethyl ester, Chinese hamster ovary cells can dissociate Sig‐1R from BiP and bind to IP3Rs, thereby reducing the degradation of IP3Rs and promoting the transfer of Ca^2+^ to mitochondria by enhancing the structural integrity of MAM. This regulatory network plays a protective role in maintaining calcium homeostasis under ERS [119]. In summary, MAM plays a pivotal role in tumorigenesis, progression, and treatment resistance by regulating Ca^2+^ signaling transduction, apoptotic protein activity, and organelle interactions. Its components can serve as novel therapeutic targets.

In conclusion, the function of MAM is to maintain cell homeostasis, not to promote survival or death in one direction. Whether it is enhanced or destroyed, it will cause the disorder of intracellular balance, thus starting the apoptosis process. In terms of mechanism, enhanced MAM‐induced apoptosis is mainly related to Ca^2+^ overload, leading to mitochondrial ΔΨm collapse, Cyt *c* release, and activation of classic intrinsic apoptosis pathway, similar to excitotoxicity; the apoptosis caused by destroying mam is due to the inhibition of energy metabolism caused by insufficient Ca^2+^ signal, which leads to the decline of the ability of cells to adapt to stress. Different apoptosis outcomes may be related to the different sensitivity of different types of cells to energy metabolism and calcium signals, as well as the different components and corresponding functions of MAM. At the same time, the stress environment of cells will also affect the outcome. For example, ERS agent will make cells in a strong stress state, and enhancing MAM can amplify the proapoptotic signal. For cells in a highly metabolic‐dependent state, destroying MAM will lead to the reduction of ATP synthesis, cause energy crisis, and then induce cell apoptosis, which needs further research and discussion.

### 5.2 Interaction Between Mitochondria and LDs Contributes to Apoptosis

**FIGURE 5:**
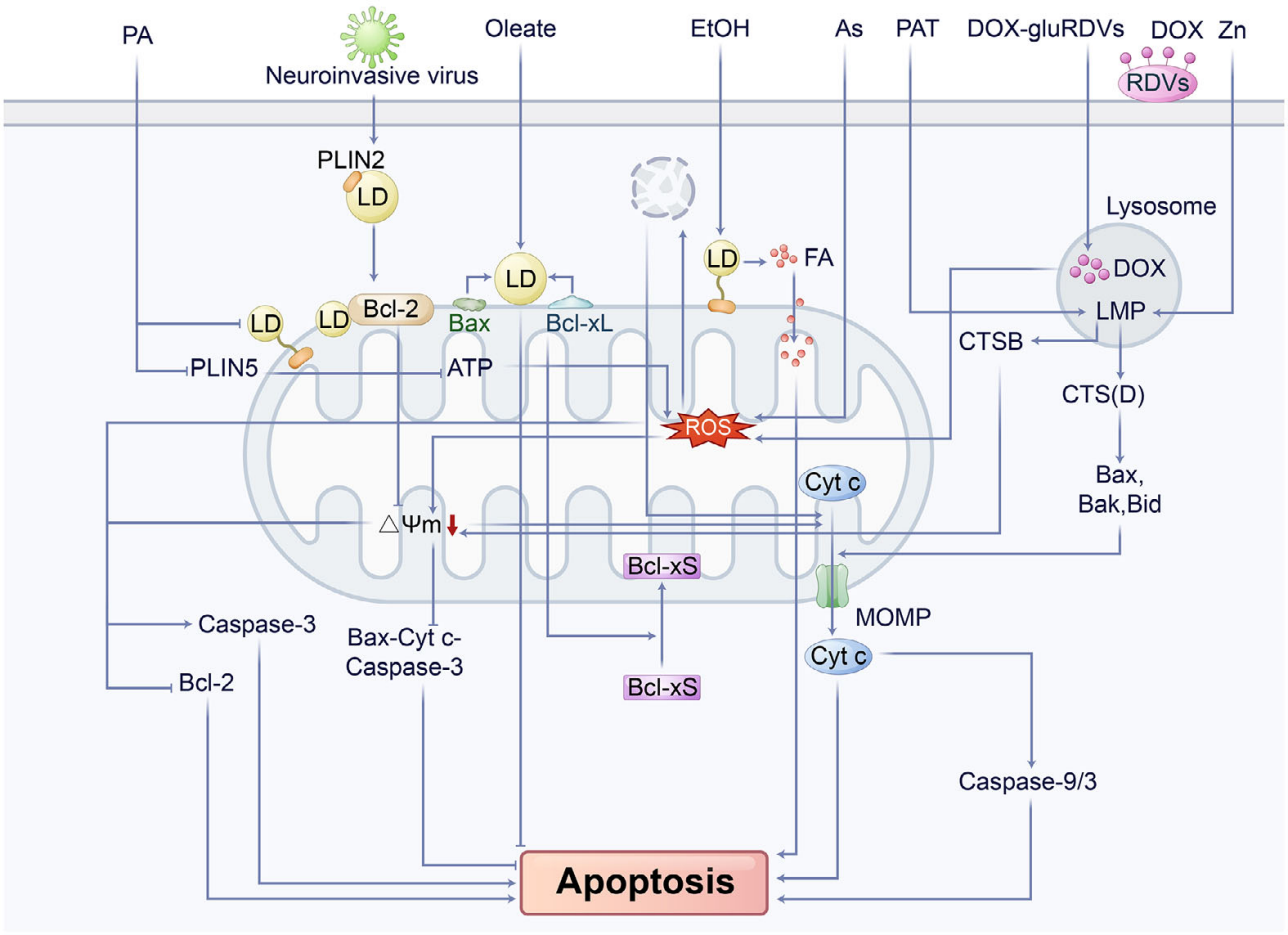
The role of mitochondria and LD interaction in apoptosis. LD and lysosomes interact with mitochondria and participate in the process of cell apoptosis. PA treatment reduces LD–Mito contacts in cells accompanied by downregulation of PLIN5 expression, which subsequently downregulates Bcl‐2 and activates Caspase‐3, ultimately inducing apoptosis. EtOH‐induced excessive LD–Mito contacts can enhance FA flux and exacerbate lipotoxicity. Neuroinvasive viruses can utilize LD surface protein PLIN2 to restore mitochondrial membrane potential, thereby preventing apoptosis in infected cells. Additionally, oleate can induce the translocation of Bax and BCL‐xL from mitochondria to LD, thereby reducing oxidative damage. Zinc exposure to cells induces LMP, which triggers MOMP, leading to Cyt *c* release and activation of the Caspase‐9/3 cascade, ultimately resulting in apoptosis. As treatment can reduce ΔΨm and disrupt lysosomal membrane integrity in cells, thereby enhancing Cyt *c* release to induce apoptosis. PAT induces LMP and CTSB release in cells, leading to the breakdown of ΔΨm and mitochondrial Cyt *c* release, ultimately triggering apoptosis. Additionally, Dox‐gluRDVs can deliver Dox into lysosomes, resulting in ΔΨm loss and Caspase‐3 activation, consequently inducing apoptosis. LD, lipid droplet; PA, palmitic acid esters; LMP, lysosomal membrane permeabilization; ROS, reactive oxygen species; As, arsenic; EtOH, ethanol; PAT, patulin; DOX, doxorubicin; RDVs, red blood cell‐derived vesicles; Bax, BCL‐2‐associated X protein; Bcl‐2, B cell lymphoma‐2; Bak, BCL‐2 antagonist/killer 1; Bid, BH3‐interacting domain death agonist; Bcl‐xL, B‐cell lymphoma‐extra‐large; Bcl‐xS, Bcl‐x short‐isoform; MOMP, mitochondrial outer membrane permeabilization; Cyt *c*, cytochrome *c*; FA, fatty acid; CTSB, cathepsin B; CTS(D), cathepsin D; PLIN2, perilipin 2; ΔΨm, membrane potential.

LD are organelles within cells that store energy in the form of neutral lipids, which can assist in mitochondrial energy supply and serve as backup energy in the event of mitochondrial dysfunction [120]. LD–mitochondria (LD–Mito) contact is an important node in lipid metabolism, and the interaction between LD and mitochondria facilitates the direct transport of fatty acids (FA) from LD to mitochondria during lipolysis, thereby preventing the cytotoxicity caused by FA accumulation (Figure 5) [121]. A study found that in neonatal rat ventricular myocytes treated with palmitic acid, LD–Mito interactions are reduced, accompanied by downregulation of PLIN5 expression. This leads to impaired mitochondrial β‐oxidation and decreased ATP production, resulting in a reduction of mitochondrial ΔΨm and an increase in ROS; consequently, the downregulation of Bcl‐2, activation of Caspase‐3, and induction of apoptosis occur, contributing to the development of lipotoxic cardiomyopathy [122]. In the human ethanol‐induced cardiotoxicity model, excessive exposure to LD–Mito can enhance FA flux and exacerbate lipotoxicity, suggesting that the dynamic equilibrium of LD–Mito exposure is crucial for maintaining cell survival [123]. LD–Mito is also involved in virus replication. In viral infectious diseases, the neuroinvasive virus can use the LD surface protein PLIN2 to restore mitochondrial potential, interact with the antiapoptotic protein Bcl‐2, and then interrupt the apoptosis pathway of Bax–Cyt *c*–Cassase‐3, thus avoiding apoptosis of infected cells. This mechanism provides protection for persistent viral infection [124]. In addition, LD can clear harmful substances on mitochondrial OMM and alleviate oxidative stress in cancer cells [125]. Adding oleate to human liver cancer cells treated with apoptosis inducer staurosporin can induce the translocation of Bax and Bcl‐xL from mitochondria to LD, reducing oxidative damage. However, it also induces the translocation of Bcl‐xS to mitochondria, suggesting the existence of cell‐type‐specific regulation in this process [126]. In summary, the LD–Mito interaction plays a “metabolic switch” role in cell apoptosis by regulating FA metabolism, oxidative stress response, and apoptotic protein localization. The disruption of its dynamic balance can lead to heart disease, viral infection, and tumor progression, providing potential targets for related treatments.

### 5.3 Interactions Between Mitochondria and the Nucleus Induce Apoptosis

**FIGURE 6:**
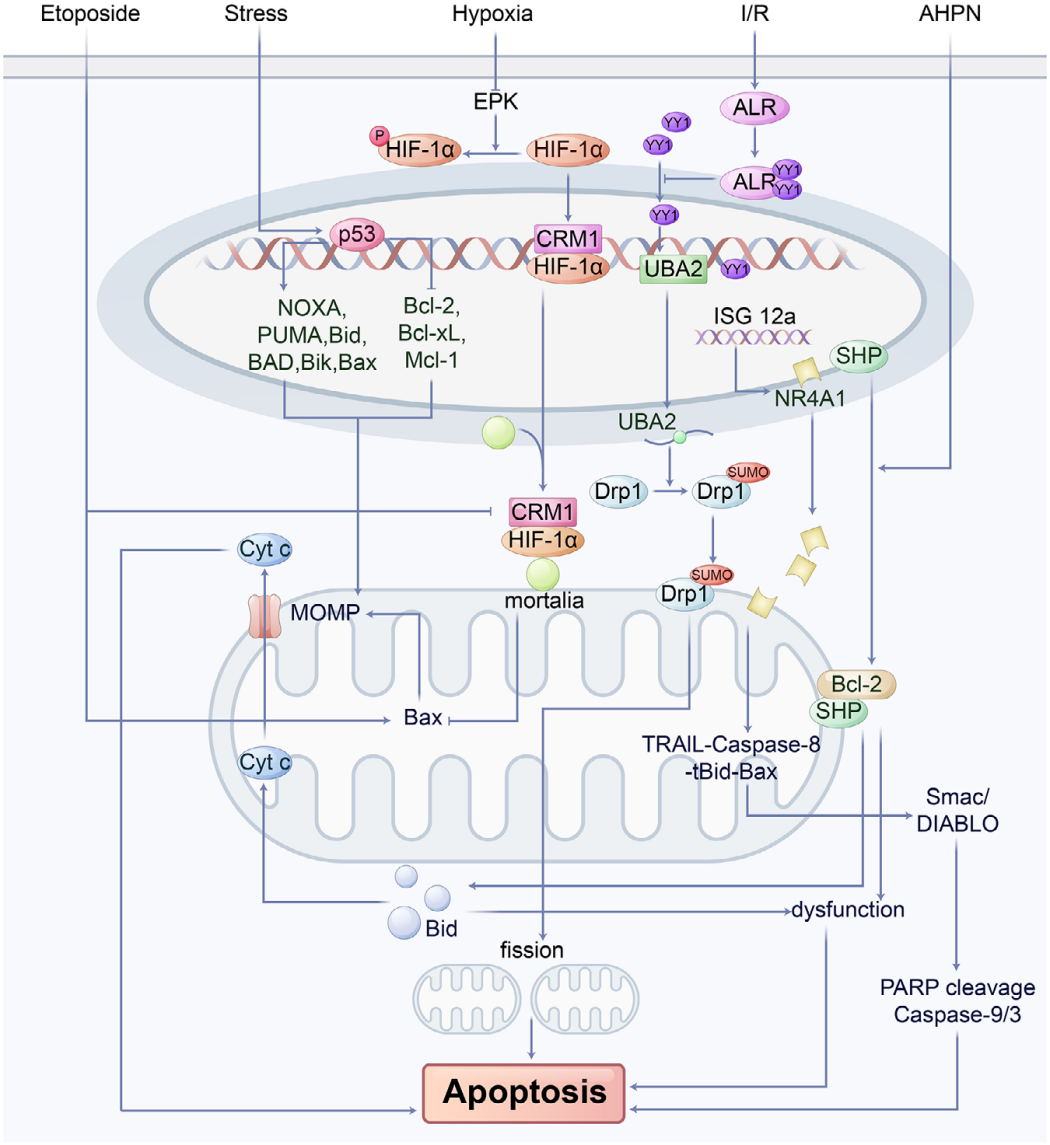
The role of mitochondria–nucleus interaction in apoptosis. Under stress conditions, nuclear p53 transcription is activated, promoting mitochondrial MOMP and Cyt *c* release, thereby inducing apoptosis. ALR can bind to the transcription factor YY1, inhibiting its nuclear translocation, which subsequently blocks Drp1‐mediated mitochondrial fission, protecting cells from IRI‐induced apoptosis. AHPN induces nuclear receptor SHP translocation to mitochondria, causing mitochondrial dysfunction and the release of Bid, promoting the release of Cyt *c*, thereby inducing cell apoptosis. Under hypoxic conditions, unmodified HIF‐1a binds to CRM1 and exits the nucleus, anchoring to mitochondria via mortalin to inhibit MOMP, thereby reducing apoptosis. Etoposide can dissociate HIF‐1α from mitochondria, thereby restoring cellular sensitivity to apoptosis. Moreover, high expression of ISG 12a promotes the translocation of nuclear receptor NR4A1 from the nucleus to the cytoplasm, subsequently targeting mitochondria, which facilitates the release of Smac/DIABLO from mitochondria, thereby enhancing PARP cleavage and Caspase‐9/3 activation, ultimately promoting apoptosis. I/R, ischemia/reperfusion; MOMP, mitochondrial outer membrane permeabilization; Cyt *c*, cytochrome *c*; AHPN, retinoid 6‐[3‐(1‐adamantyl)‐4‐hydroxyphenyl]‐2‐naphthalene carboxylic acid; EPK, extracellular signal‐regulated kinase; HIF‐1α, hypoxia‐inducible factor‐1alpha; ALR, augmenter of liver regeneration; YY1, yin‐yang‐1; NOXA, phorbol‐12‐myristate‐13‐acetate‐induced protein 1; PUMA, p53‐upregulated modulator of apoptosis; Bid, BH3‐interacting domain death agonist; Bad, Bcl‐2‐associated agonist of cell death; Bik, BCL‐2‐interacting killer; Bax, BCL‐2‐associated X protein; Bcl‐2, B cell lymphoma‐2; Bcl‐xL, B‐cell lymphoma‐extra‐large; Mcl‐1, myeloid cell leukemia 1; CRM1, chromosomal region maintenance 1; UBA2, ubiquitin activating enzyme 2; Drp1, dynamin‐related protein 1; NR4A1 NR4A member 1; ISG 12a, interferon stimulated gene‐12aSHP small heterodimer partner; TRAIL, tumour necrosis factor (TNF)‐related apoptosis‐inducing ligand; Smac/DIABLO, second mitochondria‐derived activator of caspase; PARP, poly (ADP‐ribose) polymerase.

The nucleus can affect mitochondrial function by regulating gene transcription, and some proteins can regulate cell apoptosis by translocating from the nucleus to mitochondria (Figure 6). P53 has tumor suppressive activity. Under stress conditions, activated nuclear p53 transcription can activate the expression of proapoptotic Bcl‐2 family genes (*NOXA*, *PUMA*, *BID*, *BAD*, *BIK*, *BAX*), while inactivating the expression of antiapoptotic Bcl‐2, Bcl‐xL, and Mcl‐1, promoting mitochondrial MOMP and Cyt *c* release, and inducing cell apoptosis [127]. Research has found that the SUMO modification of Drp1 is a key determinant of its mitochondrial localization, which induces mitochondrial fission. In the IRI models of humans and mice, ALR inhibits its nuclear entry by binding to transcription factor YY1, thereby downregulating the transcription of UBA2, inhibiting the SUMOylation modification of Drp1, blocking Drp1‐mediated mitochondrial division, maintaining mitochondrial integrity, and preventing IRI‐induced cell apoptosis [128].

In addition, some proteins can regulate cell apoptosis by translocating from the nucleus to the mitochondria. Retinoid 6‐[3‐(1‐adamantyl)‐4‐hydroxyphenyl]‐2‐naphthalene carboxylic acid induces nuclear receptor SHP translocation to mitochondria in liver cancer cells, disrupting mitochondrial function and releasing Bid through interaction with Bcl‐2, promoting Cyt *c* release, and inducing cell apoptosis. This process is regulated by the interaction between HNF4 α and the N‐terminal domain of SHP [129, 130]. In addition, under hypoxic conditions, ERK activity decreases in HeLa cells, making it impossible to phosphorylate HIF‐1α. Subsequently, unmodified HIF‐1α binds to CRM1 and leaves the nucleus, anchoring mitochondria through mortalin to inhibit Bax recruitment and Bax‐mediated MOMP to promote apoptosis escape [131]. Under normoxic stress conditions (etoposide), HIF‐1α dissociates from mitochondria and restores apoptosis sensitivity through Bax mitochondrial localization [115]. In addition, the highly expressed ISG 12a can promote the nuclear receptor NR4A1 in liver cancer cells to translocate from the nucleus to the cytoplasm, where it targets mitochondria and induces conformational changes in Bcl‐2, converting it into a proapoptotic molecule. This enhances the TRAIL–Caspase‐8–tBid–Bax cascade, facilitates the release of SMAC/DIABLO from mitochondria, increases PARP cleavage, activates Caspase‐9 and Caspase‐3, and ultimately promotes apoptosis [132, 133]. Some studies have also found that the nuclear receptor Nur77 translocates from the nucleus to the mitochondria, exposing the BH3 domain of Bcl‐2 and transforming it from an antiapoptotic molecule to a proapoptotic molecule [134].

### 5.4 Interactions Between Mitochondria and Lysosomes Promote Apoptosis

Lysosomes are organelles that can degrade large molecules through autophagy and endocytosis, including various peptidases [135]. Mitochondria and lysosomes cooperate to regulate apoptosis signaling pathway, mainly through the release of lysosomal membrane permeabilization (LMP) and cathepsin to cause mitochondrial dysfunction, which plays a core role in cell apoptosis (Figure 5) [136]. Exposure of zinc to PK‐15 cells induces LMP, releasing tissue protease (CTS) D into the cytoplasm, which in turn activates Bax, Bid, and Bak proteins, triggering mitochondrial MOMP. This leads to the release of Cyt *c* and activation of the Caspase‐9/3 cascade reaction, ultimately forming a lysosome–mitochondrial axis‐mediated apoptotic pathway [137]. In rat pancreatic β cells treated with 10 µM arsenic, ROS levels significantly increased, followed by a decrease in mitochondrial ΔΨm (MMP) and disruption of lysosomal membrane integrity. This led to enhanced release of Cyt *c* and induction of apoptosis, which may contribute to an increased risk of diabetes [138]. The lysosome–mitochondrial pathway, which induces cell apoptosis, has gradually become a target for anticancer. For example, treatment of liver cancer HepG2 cells with patulin can induce the release of LMP and CTSB, leading to the breakdown of ΔΨm and mitochondrial Cyt *c* release, and inducing cell apoptosis [139]. In addition, a novel nanocarrier composed of red blood cell‐derived vesicles connected to the surface of DOX to form DOX glucose‐red blood cell‐derived vesicles can effectively deliver DOX to lysosomes, leading to excessive accumulation of DOX and promoting the excessive production of mitochondrial ROS, followed by loss of MMP and activation of Caspase‐3, inducing cell apoptosis; this mechanism exhibits excellent anticancer activity in subcutaneous melanoma B16F10 carrying mice [140]. In summary, mitochondria and lysosomes synergistically regulate the apoptotic signaling pathway, mainly through the release of LMP and tissue proteases to cause mitochondrial dysfunction, playing a central role in cell apoptosis. The in‐depth study of their interaction mechanism not only provides a new perspective for revealing the pathological process of diseases (such as diabetes and cancer) but also lays a theoretical foundation for developing new therapeutic strategies targeting the interaction network of organelles.

In summary, mitochondria, as the core hub of cells, build a highly integrated dynamic “signal network” with ER, LD, nucleus, and lysosome through physical contact and molecular signals. First, mitochondria and ER are coupled at MAMs to precisely regulate calcium signaling and coordinate stress response; at the same time, it acts directly with LD to dynamically regulate the supply of FA and energy generation to prevent lipid toxicity; in addition, mitochondria communicate with nucleus in two‐way, affecting gene transcription and protein expression; and lysosome through contact site linkage, coupling energy perception and mitochondrial autophagy, to achieve quality control. These interactions are not isolated, but form a close network, enabling mitochondria to integrate energy state, metabolic demand and stress signals, and finely and cooperatively regulate the initiation and implementation of apoptosis. Understanding this complex organelle interaction network is of great significance for revealing the mechanism of related diseases and developing new treatment strategies.

## 6 Therapeutics Targeting Mitochondria to Modulate Apoptosis

As the core regulatory hub of cell apoptosis, the targeting of mitochondrial structure and function provides a key breakthrough for the intervention of a variety of diseases. In preclinical research, we can regulate the treatment strategy of cell apoptosis by accurately intervening in different dimensions of mitochondria (Table **1**). While this review lists the currently available clinical trials (Table **2**), the paucity of such studies highlights a critical need for further investigation.

**TABLE 1:** Preclinical therapeutic strategies targeting mitochondria to modulate apoptosis.

Category	Strategy/Drug	Animal/cell	Model/disease	Mechanism	Target	Effects	References
Mitochondrial morphology	Metformin	SD rats	Pre‐eclampsia	Bax, Bak and AIF↓; Bcl‐2↑; MOMP/Cyt *c* ↓	MOMP/Cyt *c*	Inhibit apoptosis	[141]
	Modified *Xiaoyao San*	Male C57 mice	Major depressive disorder	Bcl‐2↑; Bax↓; MOMP/Cyt *c* ↓; transfer AIF from mitochondria to nucleus	MOMP/Cyt *c*		[142]
	Phosphocreatine	Male SD rats	DOX‐induced nephrotoxicity	Eliminate ROS; reduce mtDNA and ETC damage; Bax↓ and Bcl‐2↑; MOMP/Cyt *c*/Caspase‐3↓	MOMP/Cyt *c*/Caspase‐3		[143]
	Carbonyl cyanide 4‐(trifluoromethoxy) phenylhydrazone	porcine proximal tubular epithelial cell line (LLC‐PK1)	Vancomycin‐induced nephrotoxicity	Reverse ROS production; maintain mitochondrial Complex I function; restore ATP production; MOMP/Cyt *c*/Caspase‐3/7↓; prevent cardiolipin peroxidationfrom	MOMP/Cyt *c*/Caspase‐3/7		[144]
	Shikonin	Human oligodendroglioma cell line Hs683	Glioma tumor	Promote Bax/Bak; MOMP/Cyt *c*/Caspase‐3↑	MOMP/Cyt *c*/Caspase‐3	Promote apoptosis	[145]
	TOS‐TPP‐OBT‐NP	HeLa cell, drug‐resistant triple negative breast cancer cell	Cancer	Bcl‐2↓; MOMP/Cyt *c*/Caspase‐3/9↑	MOMP/Cyt *c*/ Caspase‐3/9		[146]
	Indomethacin V and ibuprofen derivatives containing triarylphosphine moiety	MCF7 cells	Breast cancer	Induce mitochondrial damage, produce ROS; Bcl‐2↓; MOMP/Cyt *c*/Caspase‐3↑	MOMP/Cyt *c*/Caspase‐3		[147]
	Andro and Taxi	HeLa cells	Cervical cancer	p‐JNK/Bim pathway↑; promote the conversion of Bid to tBid; release Cyt *c* and AIF; chromatin condensation↑; MOMP, Caspase↑	MOMP; Cyt *c*; AIF		[148]
Mitochondrial dynamics	Metformin	Organ of corti 1 cells, mice	Sensorineural hearing loss	AMPK↑; the ratio of LC3II to LC3I↑; p62↓; activate mitochondrial autophagy; alleviate mitochondrial dysfunction; Cyt *c*/Caspase‐3↓	Cyt *c*/Caspase‐3	Inhibit apoptosis	[149]
	Mitochondrial acid 5	Mouse microglia BV‐2 cells	Neuroinflammation	MAPK/ERK/yap/BNIP3↑; mitochondrial autophagy↑; cellular oxidative stress↓; Caspase‐9↓	Caspase‐9		[150]
	Ligustilide	PC12 cells	Spinal cord injury	BNIP3–LC3 interaction↑; mitochondrial autophagy↑; mitochondrial dysfunction ↓			[151]
	Scutellarin	Mouse hippocampal neuron HT22 cells	Ischemic stroke	PINK1↑, Parkin↑, LC3↑, and BECN1↑, and degradate p62; mitochondrial autophagy↑; mitochondrial dysfunction↓; Bax↓, Cyt *c*↓, Caspase‐3↓, Bcl‐2↑	Bax; Cyt *c*; Caspase‐3; Bcl‐2		[152]
	Salidroside	Mouse liver cell AML‐12	Liver injury induced by a‐Amanitin poisoning	PINK1/Parkin↑; LC3II↑; P62↓; mitochondrial autophagy↑; Caspase‐3 and PARP↓	Caspase‐3 and PARP		[153]
		Primary neurons, SD rats	Spinal cord ischemia–reperfusion injury	Recover ROS‐mediated ΔΨm collapse; PINK1/Parkin↑; LC3‐B↑; p62 and Tomm20↓; mitochondrial autophagy↑; mitochondrial dysfunction↓; maintain mitochondrial homeostasis			[154]
	Quercetin	Mouse liver cell line AML12, AIF male C57BL/6J mice	ALF	PPARγ/PGC‐1α/NF‐κB/SIRT1/PINK1/Parkin↑; mitochondrial autophagy↑; MMP and ROS↓; Caspase‐3↓; Bcl‐2↑	MMP; ROS; Caspase‐3; Bcl‐2		[155]
	Naringin	Male C57BL/6J mice	Intervertebral disc degeneration	SIRT3/FOXO3a/Parkin↑; mitochondrial autophagy↑; clear ROS↑; NLRP3 inflammasome (NLRP3, ASC, pro‐Caspase‐1)↓; Caspase‐1↓	NLRP3; ASC; pro‐Caspase‐1 and Caspase‐1		[156]
	Bacopa monnieri	Immortal primary fetal astrocytes	Nervous system disease	PINK1/Parkin↑; mitochondrial autophagy↑; clear ROS↑; Caspase‐3/7↓	ROS; Caspase‐3/7		[157]
	α‐Asarone	PC‐12 cells	Diabetes encephalopathy	PINK1/Parkin↑; mitochondrial autophagy↑; Bax↓; Caspase‐3↓; Bcl‐2↑	Bax; Caspase‐3; Bcl‐2		[158]
	Urolithin A	Male C57BL/6 mice, H9C2 cells	DOX‐induced cardiac toxicity	Ambra1↑; LONP1 degradation↓; PINK1/Parkin↑; mitochondrial autophagy↑; clear damaged mitochondria↑			[159]
	Selenomethionine	Parkin gene knockout mouse	Fluoride‐induced liver injury	PINK1↓; p62↓; LC3↓; mitochondrial autophagy↓; Bax and Caspase‐3↓	Bax; Caspase‐3		[160]
	Diosgenin	SD rats	Aristolochic acid nephropathy	Drp1 and MFN2↑; mitochondrial dynamics disorders↓			[161]
	Klotho protein	H9c2 cells, C57BL/6 mice	DOX‐induced cardiac toxicity	Drp1↓; mitochondrial fission↓; Caspase‐3↓	Caspase‐3		[162]
	PPARγ agonist troglitazone	HK2 cells, adult male SD rats	Calcium oxalate kidney stones				[163]
	Diallyl trisulfide	human umbilical vein endothelial cells	Vascular injury caused by diabetes	AMPK↑; Drp1↓; mitochondrial fission↓; Cyt *c*↓, Bax↓, Caspase‐3↓	Cyt *c*; Bax; Caspase‐3		[164]
	1,8‐Cineole	Male C57BL/6 mice, β‐cell	Type 2 diabetes	Drp1 and Fis1↓; MFN1/2↑; mitochondrial dysfunction↓			[165]
	Alantolactone	HepG2 cells	HCC	PINK1/Parkin↓; mitochondrial autophagy↓		Promote apoptosis	[166]
	Morusin	Human HCC cell lines HepG2, Hep3B, and SK‐HEP‐1, BALB/c male nude mice		ROS production↑; ΔΨm and ATP↓; LC3II/LC3I ratio↑; PINK1/Parkin↑; p62↓; mitochondrial autophagy↑; Caspase‐3/9↑ and Bax↑; Bcl‐2↓	Caspase‐3/9 and Bax; Bcl‐2		[167]
	δ‐Pentabetaine	Human colorectal adenocarcinoma SW480 and SW620	CRC	COX‐1 V/SIRT3/PINK1/Parkin/LC3B↑; mitochondrial autophagy↑; Caspase‐3/9and PARP↑	Caspase‐3/9; PARP		[168]
	Rhopaloic acid A	Human oral squamous cell carcinoma cell lines HSC‐3 and Ca9‐22	Oral squamous cell carcinoma	JNK/BNIP3/Nix↑; mitochondrial autophagy↑; MMP and Bcl‐2↓; Cyt *c*/Caspase‐3↑	MMP and Bcl‐2; Cyt *c*/Caspase‐3↑		[169]
	Hypoxia‐activated prodrug CHD‐1	HeLa cells, Male BALB/c nude mice	cervical cancer	Cause MMP breakdown and mitochondrial morphological damage; LC3‐II/I ratio↑; p62↓; mitochondrial autophagy↑; Caspase‐3 and PARP1↑	Caspase‐3; PARP1		[170]
	Resveratrol	DLD1, HeLa, and MCF‐7 cells	Cancer	miR‐326PKM2↑; Drp1 and Fis1↑; mitochondrial dysfunction↑			[171]
	Bcl‐2/Bcl‐xL inhibitor ABT737	Cisplatin‐resistant cloned human OC cell line SKOV3/DDP	Cisplatin‐resistant OC	Drp1↑; mitochondrial fission/Cyt *c*/Caspase‐3/9↑	Cyt *c*/Caspase‐3/9		[172]
	2‐Methoxyestradiol	OS 143B cells	Osteosarcoma	Drp1 and Bax↑; mitochondrial fission/Cyt *c* ↑	Cyt *c*		[173]
	Baicalin	Lung cancer cell lines (A549, NCI‐H1299, and LLC)	Lung cancer	AMPK‐Drp1↑; mitochondrial fission/Cyt *c*/Caspase‐3/9↑	Cyt *c*/Caspase‐3/9		[174]
	*Qidongning*	A549, NCI‐H460, and LLC, male C57BL/6 mice		p53, Drp1 and Fis1↑; MFN1, MFN2 and OPA1↓; mitochondrial fission↑; MMP loss↑; ATP depletion↑			[175]
	Withaferin A	MDA‐MB‐231 and MCF‐7 cells	Breast cancer	Drp1, MFN1, MFN2 and OPA1‐L↓; OPA1‐S↑; disrupts normal mitochondrial cristae structures; Cyt *c* ↑	Cyt *c*		[176]
	Benzyl isothiocyanate			Bak‐Mfn1↑; Bak‐MFN2↓; p‐Drp1(S616), Drp1, Fis1, MFN1, MFN2 and OPA1↓; dysregulate mitochondrial fusion and fission			[177]
	JQ1and OTX015	MDA‐MB231, Hs578t, and BT549 cells	TNBC	Drp1↓; dysregulate mitochondrial fusion and fission			[178]
	Baicalein or Baicalein combination with 4‐hydroxytamoxifen	MCF‐7TR, T‐47DTR, ZR‐75‐1TR, BT‐474TR	Tamoxifen‐resistant breast cancer	Drp1 and Fis1↑; mitochondrial fission↑; Cyt *c*/Caspase‐9/3↑	Cyt *c*/Caspase‐9/3		[179]
mtDNA	Baicalin	Primary macrophages, mouse J774A.1 macrophage line, liver Kupffer cells, C57BL/6J mice	Inflammatory diseases	mtROS↓; mtDNA oxidation↓; block PANoptosome		Inhibit apoptosis	[180]
	Anti‐RET reagent 1‐methoxyPMS or dimethyl fumarate	Mouse macrophage lines J774A.1 and RAW 264.7, C57BL/6J mice		RET↓; NAD^+^ ↑; mtROS↓; mtDNA oxidation↓; block PANoptosome			[181]
	Fluorescent ligand SF3 derived from diphenylaminothiophene	4T1 cells	TNBC	mtDNA transcription and replication↓; disrupt mitochondrial respiratory chain; ATP↓; MMP↓; ROS↑; induce mitochondrial dysfunction; Bax and PARP↑; Bcl‐2↓	ATP; MMP; ROS; Bax and PARP; Bcl‐2	Promote apoptosis	[182]
Mitochondria–ER	Vitamin E succinate	Mouse melanoma B16F1 cells	Melanoma	GRP75↑; IP3R–GRP75–VDAC complex↑; MAM↑; lead Ca^2+^ overload	MAM	Promote apoptosis	[183]
	Paclitaxel	HeLa, UWB1.289	OC	BRCA1↑; recruit to ER; IP3R↑; MAM↑; lead Ca^2+^ overload; Caspase‐3↑	MAM; Caspase‐3		[102]
	PDI small molecule inhibitor PS89 combined with cell inhibitors	Jurkat, vincristine‐resistant CEM, CCRF‐CEM, HL‐60, ALL PDX cells	Acute leukemia	BAP31 is cleaved into proapoptotic p20 BAP31; promote Ca^2+^ release; ΔΨm↓; Caspase‐3↑	ΔΨm; Caspase‐3		[184]
	Ferulic acid	H9c2 cells	DCM	PACS2, IP3R2↓, FUNDC1 and VDAC1↓; MAM↓; Ca^2+^ overload↓	MAM	Inhibit apoptosis	[88]
	Capsaicin	Db/db podocytes, mice	DKD	Activate TRPV1 and AMPK, FUNDC1↓; MAM↓; Ca^2+^ overload↓			[94]
	ACh	Human umbilical vein endothelial cells	Hypoxia/reoxygenation injury	PI3K/Akt↑; VDAC1/Grp75/IP3R1 complex and MFN2↓; MAM↓; Ca^2+^ overload↓			[185]
Mitochondria–LD	ACh	Neonatal rat ventricular cardiomyocytes	Palmitate toxicity cardiac	PLIN5‐mediated LD–mitochondria↑; restore β‐oxidation metabolism; ATP production↑; ΔΨm ↑ and ROS↓; restore mitochondrial function; Bcl‐2↑ and Caspase‐3↓	Bcl‐2 and Caspase‐3	Inhibit apoptosis	[122]
	Ezetimibe	Immortalized AS podocytes	AS	LD–mitochondria↑; transfer FA; lipid toxicity↓; mitochondrial function↑			[186]
Mitochondria–nucleus	1,8‐Cineole	Male C57BL/6 mice, β‐cell	Type 2 diabetes	Adjust SIRT1; Nrf2/ARE↑; HO‐1 and Prdx‐1↑; ROS↓; oxidative stres damage and OXPHOS defection↓; MMP↑; mitochondrial dysfunction↓	SIRT1; Nrf2/ARE; HO‐1 and Prdx‐1; ROS; MMP	Inhibit apoptosis	[165]
	Astragaloside IV	Immortalized mouse podocytes, C57BL/6J mice	DKD	Dissociate Keap1–Nrf2; facilitate Nrf2 translocation to the nucleus; ARE, NRF‐1, PGC‐1α, and TFAM↑; ETC complex, ATP production, and mtDNA↑	ARE; NRF‐1; PGC‐1α; TFAM; ETC complex; ATP and mtDNA		[187]
	Calycosin	H9C2 cardiomyocytes, male Balb/c	Triptolide‐induced cardiac toxicity	NRF1 transcription↓; disrupt Nrf2‐Keap1; promote Nrf2, and interact with PGC‐1α; TOM20 and TFAM; maintain mitochondrial structure and mtDNA	NRF1; TOM20 and TFAM		[188]
	Phosphocreatine	Male SD rats	DOX‐induced nephrotoxicity	Nrf2/PGC‐1α↑; enhance nuclear transcription; maintain DOX homeostasis and mitochondrial integrity			[143]
	Daphnetin	HepG2 cells, ALF mouse	ALF	Degradate Keap1; Nrf2/ARE/Trx‐1↑; JNK and ASK1 phosphorylation↓; Cyt *c*, Caspase‐3 and p53 activation↓	Nrf2/ARE/Trx‐1; JNK and ASK1; Cyt *c*; Caspase‐3 and p53		[189]
	Syzygium cumini	Male albino Wistar rats, HEK‐293 cell	DKD	Keap 1↓; induce Nrf2 translocation to the nucleus; Bcl‐2 ↑; Bax; CHOP, Drp1, Cyt *c*/Caspase 3/7↓	Bcl‐2; Bax; CHOP; Drp1; Cyt *c*/Caspase 3/7		[190]
	Shen Shuai II Recipe	Male SD rat	Chronic kidney disease	The transcription of p53↓; PUMA and Bax↓; translocation and activation of p53↓; the interaction between p53 and Bcl‐xL and Bcl‐2↓; MOMP/Cyt *c*/Caspase‐3↓	p53; PUMA and Bax; Bcl‐xL and Bcl‐2; MOMP/Cyt *c*/Caspase‐3		[191]
	Lentinan	C57BL/6 mice, B16F10 melanoma cells	Melanoma	AKT phosphorylation↓; promote nuclear output of Nur77; convert Bcl‐2 from an antiapoptotic molecule to a proapoptotic molecule; PAPR↑	Nur77; Bcl‐2; PAPR	Promote apoptosis	[192]
	Cantharidin	HL‐60, Kasumi‐1, OCL‐AML3	AML				[134]
	CDDP	OVCAR‐3, A2780, A2780cp, CC, ES‐2	Epithelial ovarian cancer	activated P‐p53 (Ser15)↑; recruit HKII and AIF; translocate from mitochondria to the nucleus; chromatin condensation↑	HKII and AIF		[193]
	Baicalein or baicalein combination with 4‐hydroxytamoxifen	MCF‐7TR, T‐47DTR, ZR‐75‐1TR, BT‐474TR	Tamoxifen‐resistant breast cancer	HIF‐1α‐PHD2‐pVHL↑; the nuclear translocation of HIF‐1α↓; the transcription of *GLUT‐1*, *HK‐2*, *PDK1*, and *LDHA*↓; increase mitochondria and restore ROS; Cyt *c*/Caspase‐9/3↑	HIF‐1α; *GLUT‐1*; *HK‐2*; *PDK1*; *LDHA*; Cyt *c*/Caspase‐9/3		[179]
Mitochondria–lysosome	Antimalarial compound quinacline	HeyA8‐MDR, OVCAR5, OVCAR7, C13, OV2008, Hek293T cells	OC	CTSL↑; LMP↑; release CTSL into the cytosol; MOMP/Cyt *c*/Caspase‐3/9↑	CTSL; LMP; MOMP/Cyt *c*/Caspase‐3/9	Promote apoptosis	[194]
	Patulin	HepG2 cells	Liver cancer	LMP and CTSB↑; induce the breakdown of ΔΨm; MOMP/Cyt *c*/Caspase‐3↑	LMP and CTSB; MOMP/Cyt *c*/Caspase‐3		[139]
	Citreoviridin			LMP and CTSD↑; induce the breakdown of ΔΨm; MOMP/Cyt *c*/Caspase‐3↑	LMP and CTSD; MOMP/Cyt *c*/Caspase‐3		[195]
	Tubeimoside I	NCI‐H1299, NCI‐H1975 cells	Lung cancer	LMP and CTSB↑; induce Drp1‐mediated mitochondrial fragmentation, and inhibit V‐ATPase to block late autophagy flux; accumulate ROS; MOMP/Cyt *c*/Caspase‐3↑	LMP and CTSB; MOMP/Cyt *c*/Caspase‐3		[196]
	N‐heterocyclic carbene iridium (III) complex	A549 cells		LAMP‐1↓; MMP↓; lead to lysosome destruction and release CTSB; release Cyt *c*; Caspase‐3 and PARP↑	CTSB; MMP; Cyt *c*; Caspase‐3 and PARP		[191]
	Gefitinib + antibiotic salinomycin	HCT‐116, SW1116, SW480, HT‐29, LOVO, NCM460, SW1116, HCT‐116 mouse	CRC	Induce LMP and release CTSB and CTSD; Cyt *c*/Caspase‐3↑	LMP; CTSB; CTSD; Cyt *c*/Caspase‐3↑		[197]
	Tea polysaccharides	CT26		Disrupt lysosome function; release tissue proteases; Bak1, Caspase‐3/9↑; Bcl‐2 and MMP↓	Bak1; Caspase‐3/9; Bcl‐2 and MMP		[136]
	Costunolide	SK‐BR‐3 cells	Breast cancer	ROS production↑; release LMP and CTSD; MOMP/Cyt *c*/Caspase‐3↑; tBid, PARP ↑	LMP and CTSD; MOMP/Cyt *c*/Caspase‐3; tBid; PARP		[198]
	DOX‐gluRDVs	Mouse B16F10 cells, HT‐29 cells, MES‐SA cells and MES‐SA/DX5 cells, PC3 cells, TOV21G cells	Conventional cancer	Deliver DOX to lysosomes; Caspase‐3↑	Caspase‐3		[140]
	TPH/PTX	A549 cells	Drug‐resistant cancer	Complete lysosomal escape and locate in mitochondria; Bcl‐2↓; MOMP/Cyt *c*/Caspase‐3/9↑	Bcl‐2; MOMP/Cyt *c*/Caspase‐3/9		[199]
	CA074	HK‐2 cell, male C57BL/6 mice	AKI	LMP and CTSB↓; Bax/Caspase‐3/PARP↓	LMP and CTSB; Bax/Caspase‐3/PARP	Inhibit apoptosis	[200]
	Pyrroloquinoline quinone	Pancreatic beta cells (INS‐1 cells)	Type 2 diabetes	ROS↓; LMP and CTSB↓; MMP breakdown↓; Cyt *c*/Caspase‐3/9↓	LMP and CTSB; MMP breakdown; Cyt *c*/Caspase‐3/9		[201]
Mitochondria transfer and transplantation	Mitochondria transplantation	Intravenous administration of exogenous mitochondria‐treated macrophages in a murine MI model	MI	Promote polarization toward a reparative M2‐like phenotype; enhance migratory, invasive, and phagocytic capacities; facilitate tissue repair and mitigate inflammation; restrict ventricular remodeling; transfer residual mitochondria to cardiomyocytes; support energetic requirements		Inhibit apoptosis	[202]
		Coculture system consisting of MSCs and distressed cardiomyocytes	IRI	Transfer healthy mitochondria from MSCs to damaged cardiomyocytes via tunneling nanotubes; cytoprotective enzyme heme oxygenase‐1↑; promote mitochondrial biosynthesis	cytoprotective enzyme heme oxygenase‐1		[203]
		A mice model of proximal left anterior descending coronary artery ligation		Injured cardiomyocyte extracellular vesicles release damaged mitochondria to macrophages; alleviate ventricular dysfunction; activate macrophage Mertk‐mediated anti‐inflammatory responses			[204]
		Skeletal and cardiac muscle cells of IRI patients		Inject mitochondria from skeletal muscle into ischemic myocardium; increase energy synthesis and reduce oxidative stress; improve ventricular function			[205]
		Knock‐out of Drp1 gene from mice purkinje cells	Cerebellar degenerative diseases	Inject hepatic‐derived mitochondria from WT mice into the cerebellum of model mice; improve mitochondrial function; COX4↑ and mitophagy markers (Parkin and LC3B)↓; Caspase‐3↓	COX4 and mitophagy markers (Parkin and LC3B); Caspase‐3		[206]
		Swine renal cells subjected to 60 min of renal ischemia and 24 h of reperfusion following bilateral renal artery occlusion	AKI	Mitochondria enter the cell via actin‐dependent endocytosis; maintain mitochondrial homeostasis; restore ATP production; ROS levels↓; maintain mitochondrial homeostasis; energy metabolism↑	ATP; ROS		[207]
		Mouse model of Parkinson's disease induced by 1‐methyl‐4‐phenyl‐1,2,3,6‐tetrahydropyridine	Parkinson's disease	Stereotaxic inject mitochondrial capsules to injured cells; increase respiratory chain complex activity and restore mitochondrial energy production			[208]
		Dguok knockout mice	Hepatocerebral mtDNA depletion syndrome	Stereotaxic inject mitochondrial capsules to injured cells; repair hepatocyte mtDNA			[208]

*Abbreviations*: ER: endoplasmic reticulum; GRP75: glucose regulatory protein 75 kDa; MAM: mitochondria‐associated endoplasmic reticulum membranes; IP3R: inositol 1,4,5‐triphosphate receptor; VDAC: voltage‐dependent anion channel; WT: wild‐type; OC: ovarian cancer; BRCA1: breast cancer 1 protein; DCM: diabetic cardiomyopathy; PACS2: phosphofuran acidic cluster sorting protein 2; IP3R2: inositol 1,4,5‐triphosphate receptor 2; FUNDC1: FUN14 domain containing 1; VDAC1: voltage‐dependent anion channel 1; DKD: diabetic kidney disease; TRPV1: transient receptor potential vanilloid 1; AMPK: AMP activated protein kinase; ACh: acetylcholine; HUVECs: human umbilical vein endothelial cells; IP3R1: inositol 1,4,5‐triphosphate receptor 1; PI3K: phosphatidylinositol 3‐kinase; Akt: protein kinase B; PDI: protein disulfide isomerase; ALL: acute lymphoblastic leukemia; PDX: patient‐derived xenograft; BAP31: B cell receptor‐associated protein 31; p20 BAP31: 20 kDa proapoptotic fragment of BAP31; ΔΨm: mitochondrial membrane potential; PARP: poly(ADP‐ribose) polymerase; PLIN5: perilipin 5; NRVC: neonatal rat ventricular cardiomyocytes; PA: palmitic acid esters; LD: lipid droplets; β‐oxidation: beta‐oxidation; ROS: reactive oxygen species; Bcl‐2: B cell lymphoma‐2; AS: Alport syndrome; FA: fatty acid; STZ: streptozotocin; T2DM: Type 2 diabetes mellitus; Drp1: dynamin‐related protein 1; Fis1: fission 1 protein; MFN1/2: mitofusin 1/2; SIRT1: sirtuin 1; Nrf2: nuclear factor erythroid‐2‐related factor 2; ARE: antioxidant response element; HO‐1: heme oxygenase‐1; Prdx‐1: peroxiredoxin 1; OXPHOS: oxidative phosphorylation; MMP: mitochondrial membrane potential; Keap1: Kelch‐like ECH‐associated protein 1; NRF‐1: nuclear respiratory factor 1; PGC‐1α: peroxisome proliferator‐activated receptor gamma coactivator 1α; TFAM: mitochondrial transcription factor A; ETC: electron transfer chain; mtDNA: mitochondrial DNA; TOM20: translocase of outer mitochondrial membrane 20; DOX: doxorubicin; PCr: phosphocreatine; MOMP: mitochondrial outer membrane permeabilization; Bax: Bcl‐2‐associated X protein; Cyt *c*: cytochrome *c*; ALF: acute liver failure; APAP: acetaminophen; Trx‐1: thioredoxin‐1; JNK: c‐Jun N‐terminal kinase; ASK1: apoptosis signal‐regulating kinase 1; CHOP: C/EBP homologous protein; AKT: protein kinase B; Nur77: nuclear receptor Nur77; AML: acute myeloid leukemia; CDDP: cisplatin; EOC: epithelial ovarian cancer; P‐p53 (Ser15): phosphorylated p53 at serine 15; HKII: hexokinase II; AIF: apoptosis inducing factor; HIF‐1α: hypoxia inducible factor‐1α; PHD2: prolyl hydroxylase domain‐containing 2; pVHL: von Hippel‐Lindau tumor suppressor; GLUT‐1: glucose transporter protein 1; HK‐2: hexokinase 2; PDK1: 3‐phosphoinositide‐dependent kinase 1; LDHA: lactate dehydrogenase A; CKD: chronic kidney disease; PUMA: p53‐upregulated modulator of apoptosis; Bcl‐xL: B‐cell lymphoma‐extra‐large; CTSL: cathepsin L; LMP: lysosomal membrane permeabilization; Bid: BH3‐interacting domain death agonist; CTSB: cathepsin B; CTSD: cathepsin D; V‐ATPase: vacuolar‐type H^+^‐ATPase; CRC: colorectal cancer; Bak1: Bcl‐2 antagonist/killer 1; Caspase‐9: cysteine‐aspartic acid protease 9; DOX‐gluRDVs: doxorubicin‐glucose‐red blood cell‐derived vesicles; RDVs: red blood cell‐derived vesicles; TPH/PTX: TPP‐pluronic F127‐hyaluronic acid/paclitaxel; HA: hyaluronic acid; PTX: paclitaxel; HAase: hyaluronidase; LPS: lipopolysaccharide; AKI: sepsis‐induced acute kidney injury; mtROS: mitochondrial reactive oxygen species; Z‐DNA: Z‐form DNA; ZBP1: Z‐DNA‐binding protein 1; Ripk3: receptor‐interacting protein kinase 3; ASC: apoptosis‐associated speck‐like protein containing a CARD; NAD+: nicotinamide adenine dinucleotide; TNBC: triple negative breast cancer; TNF‐α: tumor necrosis factor α; MAPK: mitogen‐activated protein kinase; ERK: extracellular signal‐regulated kinase; yap: Yes‐associated protein; PINK1: phosphatase and tensin homolog‐induced kinase 1; Parkin: E3 ubiquitin ligase Parkin; LC3: microtubule‐associated protein 1 light chain 3; BECN1: beclin‐1; p62: sequestosome 1; ROS: reactive oxygen species; LC3II: microtubule‐associated protein 1 light chain 3 II; SIRT3: sirtuin 3; FOXO3a: forkhead box o3a; NLRP3: NOD‐like receptor family pyrin domain‐containing 3; HG: high glucose; HCC: hepatocellular carcinoma; COX‐1V: cytochrome *c* oxidase subunit IV; PPARγ: peroxisome proliferator‐activated receptor gamma; PE: pre‐eclampsia; TOS‐TPP‐OBT‐NP: alpha tocopherol succinate–triphenylphosphine–obatoclax–nanoparticles; AIF: apoptosis inducing factor; PGC‐1α: peroxisome proliferator‐activated receptor‐gamma coactivator‐1α; BNIP3: Bcl‐2 and adenovirus E1B 19 kDa‐interacting protein 3; NF‐κB: nuclear factor κB; Nix: BNIP3‐like; LAMP‐1: lysosomal‐associated membrane protein 1; RET: reverse electron transfer; Ambra1: autophagy/beclin‐1 regulator 1; LONP1: lon peptidase 1; PKM2: pyruvate kinase M2; miR‐326: microRNA‐326; MI: myocardial infarction; MSCs: Mesenchymal stem cells: ↑: upregulation, ↓: downregulation.

**TABLE 2:** Clinical therapeutic strategies targeting mitochondria to modulate apoptosis.

Treatment	Target	Disease	NCT No.	Status	Phases	Objectives	Research findings	References
ABT‐199/venetoclax	Induce MOMP	R/R AML	NCT01994837	Completed	Phase 2	Evaluate the efficacy and safety	The overall response rate was 19%, the drug demonstrated antitumor activity and acceptable tolerability in AML patients with adverse features.	[209, 210]
ABT‐263/navitoclax		R/R ALL and lymphoblastic lymphoma	NCT03181126	Completed	Phase 1		In patients who had received adequate prior treatment, the combination chemotherapy was well tolerated and yielded satisfactory efficacy with high CR/CRi/CRp rates.	[211, 212]
		R/R chronic lymphocytic leukemia	NCT00481091	Completed	Phase 1, Phase 2		Lymphocytosis was reduced by more than 50% in 19 of 21 patients with baseline lymphocytosis; median progression‐free survival was 25 months.	[213, 214]
TJ0113	Activae mitochondrial autophagy	Parkinson's disease	NCT06596005	Completed	Phase 2		All patients demonstrated favorable efficacy and symptom improvement, with no drug‐related serious adverse events or withdrawal reactions observed; overall safety and tolerability were good.	[215, 216]
Mitochondria transplantation	—	Cerebral ischemia	NCT04998357	Recruiting	Phase 1	A first‐in‐human‐brain trial to confirm the safety of autologous mitochondrial transplant during brain ischemia	No significant adverse events were observed with the endovascular approach during reperfusion therapy, and safety outcomes in study participants were comparable to those of matched controls who did not undergo transplantation.	[217, 218]
		Myocardial infarction	NCT05669144	Unknown status	Phase 1, Phase 2	Try to minimize the damage by transplantation of mitochondria	—	[219]
		Ischemic myocardium	NCT02851758	Recruiting	Not applicable	Try to ameliorate myocardial IRI and significantly decrease morbidity and mortality in patients requiring ECMO	—	[220]

*Abbreviations*: MOMP: mitochondrial outer membrane permeabilization;R/R: relapsed/refractory; AML: acute myelogenous leukemia; ALL: acute lymphoblastic leukemia; CR: complete remission/response; CRi: CR with incomplete hematologic recovery; CRp: CR with incomplete platelet recovery; mtDNA: mitochondrial DNA; HCC: hepatocellular carcinoma; IRI: ischemia–reperfusion injury; ECMO: extracorporeal membrane oxygenation.

### 6.1 Targeting Mitochondrial Morphology

MOMP is an important pathway for mitochondrial apoptosis, and inhibiting MOMP can alleviate cell damage and exert a protective effect. At present, some drugs have achieved therapeutic effects by targeting MOMP. Metformin, an oral antidiabetic drug, can significantly downregulate Bax, Bak, and AIF and upregulate Bcl‐2, and negatively regulate MOMP, thereby reducing apoptosis in the mitochondrial pathway of pre‐eclampsia rats [141]. At the same time, modified Xiaoyao San can improve MOMP in the hippocampus of severe depression model mice, reduce Cyt *c* release, and AIF translocation from mitochondria to the nucleus, thereby improving neuronal apoptosis [142]. Phosphocreatine directly scavenges ROS and reduces damage to mtDNA and the electron transfer chain, while downregulating Bax and upregulating Bcl‐2 to inhibit MOMP, decrease Cyt *c* release, and suppress Caspase‐3 activation, thereby alleviating DOX‐induced nephrotoxicity [143].

Meanwhile, MOMP also plays a role in cancer treatment, as shikonin activates Bax/Bak‐dependent MOMP to promote Cyt *c* release and induce apoptosis in glioma cells [145]. The triphenylphosphine‐coated nanoparticles constructed from Complex II inhibitor alpha tocopherol succinate and Bcl‐2 inhibitor obatoclax (TOS–triphenylphosphine–OBT–NP) can specifically inhibit antiapoptotic Bcl‐2 protein and trigger MOMP, providing a new approach for chemotherapy resistance [146]. Indomethacin V and ibuprofen derivatives containing triarylphosphine can induce mitochondrial damage through MOMP, and then produce ROS, which significantly inhibits the proliferation of breast cancer cells [147]. Andrographolide and PTX induce MOMP‐dependent AIF and Cyt *c* release through the JNK/Bim pathway or promote the conversion of proapoptotic protein Bid to tBid, ultimately leading to chromatin condensation and caspase cascade activation in HeLa cells, causing cytotoxic effects [148]. Notably, some Bcl‐2 inhibitors have been shown to play a critical role in hematologic malignancies by inducing MOMP, which has been confirmed in clinical trials [221, 222].

In addition, in pig proximal tubular epithelial cells, carbonylcyanide‐4‐(trifluoromethoxy) phenylhydrazone can reverse mitochondrial ROS production and mitochondrial CL peroxidation, thereby inhibiting MOMP, reducing Cyt *c* release and Caspase‐3/7 activation, while repairing mitochondrial Complex I function to restore ATP synthesis, ultimately alleviating cell damage and reducing vancomycin‐induced nephrotoxicity [144].

### 6.2 Targeting Mitochondrial Dynamics

Research has shown that mitochondrial autophagy can bidirectionally regulate cell apoptosis through different mechanisms, and its direction of action depends on the type of disease and drug molecular targets. For most drugs, inhibiting apoptosis by activating mitochondrial autophagy has become a therapeutic target. Metformin activates AMPK‐dependent mitochondrial autophagy and alleviates tert‐butyl hydroperoxide‐induced mitochondrial dysfunction and endogenous apoptosis pathways, thereby protecting auditory hair cells and alleviating sensorineural hearing loss [149]. In addition, mitochondrial acid‐5 upregulates BNIP3 through the MAPK–ERK–yap pathway to activate mitochondrial autophagy, thereby improving energy metabolism and inhibiting Caspase‐9‐dependent apoptosis, promoting neuronal survival after inflammatory injury [150]. Ligustilide reverses oxidative stress‐induced downregulation of BNIP3 and enhances mitochondrial autophagy through BNIP3–LC3 interaction, thereby reducing mitochondrial dysfunction and alleviating spinal cord injury [151]. In addition, inhibiting apoptosis by enhancing mitochondrial autophagy mediated by the PINK1–Parkin pathway is also an important mechanism by which drugs exert their effects. Scutellaria [152], salidroside [153, 154], quercetin [155], bacopa monnieri [157], α‐asarone [158], urolithin A [159], and TJ0113 [223] can all promote mitochondrial autophagy, maintain mitochondrial homeostasis, and inhibit the expression of proapoptotic proteins through the PINK1–Parkin pathway, exerting antiapoptotic effects. Additionally, in a mouse model of intervertebral disc degeneration, naringin activates the SIRT3/FOXO3a/Parkin pathway, enhancing mitochondrial autophagy and ROS clearance. This inhibits the NLRP3 inflammasome (including apoptosis‐associated speck‐like protein containing a CARD/pro‐Caspase‐1 activation and Caspase‐1‐dependent apoptosis), protects chondrocytes from oxidative stress‐induced apoptosis, and exerts antioxidant and anti‐inflammatory effects [156]. Meanwhile, the sesquiterpene lactone Alantolactone can induce apoptosis by inhibiting PINK1/Parkin‐mediated mitochondrial autophagy and has the potential for treating liver cancer [166]. On the contrary, studies have found that activating mitochondrial autophagy can also promote cell apoptosis and play a role in cancer treatment. Both the isoprenoid flavonoid compound Morusin and the emerging dietary metabolite δ‐pentetaine can activate mitochondrial autophagy mediated by the PINK1–Parkin pathway, thereby upregulating cleaved Caspase‐3/9 and exerting cytotoxic effects in liver cancer and colorectal cancer, respectively [167, 168]. Terpene compound Red Bean Acid A induces mitochondrial autophagy mediated by JNK/BNIP3/Nix, leading to loss of MMP, decreased expression of Bcl‐2, and release of Cyt *c*, activating Caspase‐3/PARP‐dependent apoptosis. RA exhibits significant anticancer activity in human oral squamous cell carcinoma models and may be a novel and effective treatment method [169]. Hypoxia‐activated prodrug CHD‐1 can induce mitochondrial autophagy through mitochondrial damage, promote the activation of proapoptotic proteins, and target the inhibition of hypoxic tumor cells [170]. Meanwhile, selenomethionine can inhibit PINK1/Parkin‐mediated mitochondrial autophagy to alleviate fluoride‐induced liver injury and reduce apoptosis [160].

In addition, precise regulation of mitochondrial fusion and fission is also an important target for disease treatment: diosgenin upregulates mitochondrial dynamics‐related proteins Drp1 and MFN2 to improve renal mitochondrial dynamics disorder induced by aristolochic acid I [161]. Klotho protein treatment [162], PPARγ agonists troglitazone [163], and diallyl trisulfide [164] can all inhibit Drp1‐mediated mitochondrial fission, thereby downregulating mitochondrial‐related apoptosis markers (such as Cyt *c*, Bax, Bcl‐2, Caspase‐3), inhibiting cell apoptosis and reducing disease‐related damage. Drugs such as resveratrol [171], Bcl‐2/Bcl‐xL inhibitors ABT‐737 [172], 2‐methoxyestradiol [173], baicalein [174], and traditional Chinese medicine Qidongning [175] upregulate mitochondrial fission factor Drp1, leading to mitochondrial fission, promoting cancer cell apoptosis, and exerting anticancer effects. In human breast cancer cells, the medicinal phytosterolide withaferin A [176], phytochemical benzyl isothiocyanate [177], and BET proteins' function using bromodomain inhibitors (BETi) [178] destroy the structure and function of mitochondria by downregulation of mitochondrial dynamics‐related proteins, thus inducing apoptosis and inhibiting the progress of breast cancer.

### 6.3 mtDNA

Abnormalities in mtDNA can participate in disease progression by affecting drug response. A study has found that mtDNA mutations in prostate cancer can weaken the apoptosis‐inducing effect of statins, while an increase in mtDNA copy number stabilizes mitochondrial membrane permeability and function, protecting hair cells from apoptosis induced by neomycin or DDP [224, 225]. For inflammatory diseases, baicalin and antireverse electron transfer reagents, such as 1‐methoxy PMS or dimethyl fumarate, can prevent mtDNA peroxidation, block the assembly of PANoptosomes (including ZBP1, RIPK3, apoptosis‐associated speck‐like protein containing a CARD, and Caspase‐8), thereby inhibiting PANoptosis and exerting a protective effect [180, 181]. In addition, the fluorescent ligand SF3 derived from diphenylaminothiophene can inhibit the mtDNA transcription and replication of triple negative breast cancer cells, destroy the mitochondrial respiratory chain, reduce the production of ATP, and then lead to the loss of MMP and the accumulation of ROS, resulting in mitochondrial dysfunction, triggering apoptosis, and has anticancer activity [182].

### 6.4 Targeting Interactions Between Mitochondria and Other Organelles

#### 6.4.1 Targeting Interactions Between Mitochondria and ER

Research has found that vitamin E succinate and PTX promote the formation of MAM, which in turn leads to mitochondrial Ca^2+^ overload, induces apoptosis, and plays a therapeutic role in cancer [102, 183]. Ferulic acid [88], capsaicin [94], and acetylcholine [185] inhibit MAM formation, alleviate Ca^2+^ overload, inhibit apoptosis, and protect cells from stress damage. In addition, the small molecule inhibitor PS89 of PDI combined with cell inhibitors cleaves BAP31 mediated by Caspase‐8 into proapoptotic p20 BAP31 at the MAM site, which then promotes ER release of Ca^2+^ and induces mitochondrial ΔΨm decrease, activates Caspase‐3, upregulates cleaved PARP, promotes cell apoptosis, and has a chemical sensitization effect [184].

#### 6.4.2 Targeting Interactions Between Mitochondria and LDs

Acetylcholine can partially promote lipid breakdown in LD and activate the interaction between LD and mitochondria through PLIN5, restoring mitochondrial β‐oxidation metabolism, increasing ATP production, thereby increasing ΔΨm and reducing ROS to restore mitochondrial function and protect cardiomyocytes from palmitate‐induced apoptosis [122]. The lipid‐lowering drug ezetimibe improves FA transport by enhancing LD mitochondrial contact, alleviating podocyte lipotoxicity and mitochondrial dysfunction in Alport syndrome [186].

#### 6.4.3 Targeting Interactions Between Mitochondria and Nucleus

Research has found that 1,8‐cineole [165], Astragaloside IV [187], calycosin [188], and creatine phosphate [143] cause Nrf2 translocation to the nucleus, which in turn promotes transcription of mitochondrial biogenesis‐related genes to alleviate mitochondrial dysfunction, inhibit cell apoptosis, and effectively alleviate disease damage. Syzygium cumini and daphnetin can also promote Nrf2 nuclear translocation, which in turn affects the expression of apoptotic proteins such as Bcl‐2, thereby preventing the release of Cyt *c* and inhibiting the process of cell apoptosis [189, 190]. In addition, lentinan and cantharidin can promote the translocation of Nur77 from the nucleus to the mitochondria, colocalized with Bcl‐2 in the mitochondria, thereby converting Bcl‐2 from an antiapoptotic molecule to a proapoptotic molecule and inducing cell apoptosis to treat malignant tumors [134, 192]. In chemotherapy‐sensitive human epithelial ovarian cancer cells, cisplatin induces inducible activation of P‐p53 (Ser15), leading to the translocation of glycolytic enzymes HKII and AIF from mitochondria to the nucleus. AIF‐mediated chromatin condensation and nuclear HKII‐P‐p53 (Ser15) formation enhance OC cell apoptosis [193]. Therefore, it can be inferred that nuclear HKII‐P‐p53 (Ser15) may be a promising prognostic biomarker [193]. Baicalein promotes the interaction of HIF‐1α with PHD2 and pVHL, and then inhibits HIF‐1α nuclear translocation, reduces glycolysis gene transcription (GLUT‐1/HK‐2/PDK1/LDHA), thus increasing the number of mitochondria and restoring ROS, helping to enhance 4‐hydroxytamoxifen‐induced Cyt *c* release and Caspase‐9/3 activation, and improving the drug resistance of breast cancer [179]. The Chinese herbal medicine Shen Shuai II Recipe can inhibit p53 nuclear transcription activity, downregulate PUMA/Bax, block p53 mitochondrial translocation, weaken Bcl‐xL/Bcl‐2 interaction, delay MOMP‐induced apoptosis inhibition, and thus delay the development of chronic kidney disease [191].

#### 6.4.4 Targeting Interactions Between Mitochondria and Lysosomes

LMP has a synergistic effect with mitochondrial apoptosis. Research has found that antimalarial compounds quinacline [194], patulin [139], citrooviridin [195], N‐heterocyclic carbene iridium (III) complex [191], combination of antibiotic salinomycin and gefitinib GEF [197], tea polysaccharides [136], costunolide [198], and traditional Chinese medicine anticancer agent Tubeimoside I [196] can all exert anticancer effects by disrupting lysosomal function, causing LMP, and subsequently releasing tissue proteases, thereby promoting mitochondrial apoptosis pathway. Meanwhile, selective CTSB inhibitors CA074 and pyrroloquinoline quinone can also inhibit LMP‐mediated mitochondrial apoptosis and alleviate disease progression [200, 201]. In addition, DOX glucose‐red blood cell‐derived vesicles deliver DOX into lysosomes, causing excessive accumulation of DOX and promoting mitochondrial ROS accumulation to induce cell apoptosis [124]. Triphenylphosphine Pluronic F127 hyaluronic acid (HA) (TPH)/PTX nanomicelles enter acidic lysosomes through macropinocytosis, causing HAase in the acidic lysosomes to degrade HA, resulting in positively charged TP/PTX nanomicelles completing lysosomal escape and ultimately locating in mitochondria [199]. By inhibiting antiapoptotic Bcl‐2, MOMP is induced, leading to the release of Cyt *c* and inducing apoptosis in lung cancer cells, demonstrating significant tumor targeting [199].

### 6.5 Mitochondria Transfer and Transplantation

Accumulating evidence in recent years indicates that mitochondria do not function solely within isolated cells; instead, they can undergo intercellular transfer via tunneling nanotubes, extracellular vesicles, gap junction channels, and cell fusion, which is tightly regulated by cellular bioenergetics, serves to enhance metabolic regulation [226]. Building on this natural phenomenon, mitochondrial transplantation has emerged as a promising therapeutic strategy, demonstrating significant potential across various disease models by delivering isolated healthy mitochondria to metabolically compromised tissues to boost ATP production, restore redox balance, and ultimately enhance cellular stress resistance while reducing apoptosis [227]. This therapeutic strategy demonstrates remarkable efficacy in cardiac tissue, where the infusion of mitochondria into the myocardium or coronary arteries in mammalian models of IRI significantly reduces infarct size and consequently improves cardiac function [202, 203, 204, 228]. Emani et al. transplanted autologous mitochondria, isolated and purified from skeletal muscle, into ischemic hearts [205]. Their findings revealed significantly improved cardiac function and a strong safety profile, representing the first successful instance of mitochondrial transplantation for treating human myocardial IRI [205]. Moreover, mitochondrial transplantation has proven effective across a spectrum of acute and chronic diseases [206]. Notably, in a mouse model mimicking cerebellar neurodegeneration, the administration of liver‐derived mitochondria significantly enhanced mitochondrial function, mitigated excessive mitophagy, and delayed apoptosis for up to 3 weeks, suggesting a promising therapeutic potential for early‐stage cerebellar neurodegenerative disorders [206]. Simultaneously, in the context of renal diseases, studies have demonstrated that mitochondrial transplantation in a swine model of IRI–AKI prevents renal tubular cell death, thereby enhancing regenerative potential [207]. However, mitochondrial transplantation currently faces several translational challenges, including delivery precision, disease heterogeneity, durability of therapeutic effects, protocol standardization, and safety concerns, which necessitate further in‐depth investigation [207]. Notably, a recent study has introduced a novel transplantation strategy that encapsulates healthy mitochondria within erythrocyte membrane‐derived vesicles to form “mitochondrial capsules,” achieving a remarkable 80% transplantation efficiency and offering a promising new avenue for the advancement of this field [208].

## 7 Perspective and Conclusion

Mitochondria are not only the core of energy metabolism, but also the key node of regulating cell apoptosis. As the regulatory core of cell energy metabolism, mitochondria are deeply involved in the precise regulation of cell apoptosis through multidimensional mechanisms. Its functional imbalance is not only closely related to a variety of pathological processes such as neurodegenerative diseases, cardiovascular diseases, and malignant tumors, but also constructs a multilevel apoptosis regulation system through the unique morphological plasticity, dynamic characteristics, genetic material regulation, organelle communication network, and other complex mechanisms. Specifically, the morphology of mitochondria (including MOMP, cristae remodeling, and lipid distribution) constitutes the structural basis of its function, and further fine regulates the release of proapoptotic factors such as Cyt *c*; the dynamic process, that is, the fission mediated by Drp1 and the fusion mediated by MFN1/2 and OPA1, dynamically reshapes the structural basis, realizes the renewal of the mitochondrial network, directly regulates its morphology, distribution, and functional integrity, and affects the activation and localization of apoptotic proteins. The mtDNA located at the core of this dynamic structure indirectly regulates apoptosis sensitivity by encoding the key subunit of respiratory chain complex and acting as a congenital immune signal molecule. This internal operation system is further integrated into the overall cell environment through the systematic interaction with other organelles: for example, the contact at MAMs realizes the efficient exchange and collaborative regulation of Ca^2+^, lipids, and apoptosis signal molecules; interaction with lysosomes regulates mitochondrial autophagy to maintain quality control; interaction with LD coordinates FA supply and energy generation; and the nucleus through gene expression, thereby affecting mitochondrial function. These interaction networks make mitochondria become the core processor for sensing internal and external stress and integrating apoptosis signals and constitute a multilevel, fine‐tuning target system.

Although the research has made important progress, there are still significant challenges in this field: first, the specific molecular mechanism and spatio‐temporal regulation of the key processes such as mitochondrial ridge remodeling, dynamic changes and mtDNA release have not been fully clarified; second, in the pathological microenvironment (such as tumors and neurodegenerative diseases), how the multidimensional regulatory mechanisms of mitochondria work together, and the dynamic network affected by cell type and microenvironment is still lack of systematic analysis; in addition, most of the existing studies focus on a single mechanism or condition, and lack a systematic research model integrating morphological dynamics, epigenetic regulation, and multiorganelle interaction, which limits our understanding of the global regulation of mitochondrial apoptosis.

Based on the above limitations, further exploration can be carried out in the following directions in the future: (1) molecular mechanism research: systematic analysis of mitochondrial morphology, dynamic changes, DNA, and its interaction mechanism in cell apoptosis, focusing on the role of key regulatory proteins and signaling pathways; (2) dynamic regulatory network: using multiomics and dynamic imaging technology, we built a dynamic data model of mitochondrial apoptosis regulation, and studied the dynamic regulatory network and its changes among mitochondrial mechanisms in different cell types and disease models; (3) clinical transformation research: explore the potential application of mitochondrial apoptosis regulation mechanism in disease treatment, such as the development of nano drug delivery system targeting mitochondria, precise drug delivery strategy based on ΔΨm gating, and the transformation of mitochondrial apoptosis mechanism into antiaging or immunotherapy; (4) application of new technology: integrating artificial intelligence, organoid and microfluidic chips, single cell analysis, and ultra‐high‐resolution imaging technology, which will further reveal the complex regulatory mechanism of mitochondria in cell apoptosis. Notably, recent studies have revealed that mitochondria are not a homogeneous entity but possess distinct functional compartments, forming specialized subpopulations dedicated to “energy supply” and “biosynthesis,” respectively. This discovery not only elucidates the adaptive mechanisms of cells under nutritional stress but also introduces a novel perspective of mitochondrial subpopulation heterogeneity, paving the way for in‐depth research into the precision of cellular metabolic regulation [229]. In conclusion, mitochondria are deeply involved in the regulation of apoptosis through multidimensional mechanisms, and its research is of great significance for understanding the mechanism of cell death and the treatment of related diseases. At the same time, future research needs to combine the exploration of basic mechanisms with clinical transformation, especially focusing on the versatility of mitochondria as the “hub of cell fate decision,” so as to provide new targets for disease treatment.

## Author Contributions

Rubin Tan and Chunxiang Zhang: conceptualization, administration, funding, supervision, original draft preparation, reviewing, and editing. Jinming Zhao and Rong Wang: original draft preparation, reviewing, and editing. Yue Shi, Wenqing Duan, Zaozao Chen, and Duo Lin: original draft preparation and editing. Yixuan Ma and Na Li: revising, reviewing, and editing. All authors read and approved the final manuscript.

## Funding

This work was supported by grants from the National Natural Science Foundation of China (81700055, U23A20398, 82030007), Noncommunicable Chronic Diseases‐National Science and Technology Major Project (2024ZD0537707), and the Natural Science Foundation of Jiangsu Province (BK20160229).

## Conflicts of Interest

The authors declare no conflicts of interest.

## Ethics Statement

The authors have nothing to report.

## Data Availability

The authors have nothing to report.
